# Zeolite Nanoparticles for Selective Sorption of Plasma Proteins

**DOI:** 10.1038/srep17259

**Published:** 2015-11-30

**Authors:** M. Rahimi, E.-P. Ng, K. Bakhtiari, M. Vinciguerra, H. Ali Ahmad, H. Awala, S. Mintova, M. Daghighi, F. Bakhshandeh Rostami, M. de Vries, M. M. Motazacker, M. P. Peppelenbosch, M. Mahmoudi, F. Rezaee

**Affiliations:** 1 Faculty of Science, University of Groningen, University Medical Center Groningen, Groningen, the Netherlands; 2 School of Chemical Sciences, University Sains Malaysia, 11800 USM, Malaysia; 3 Department of Plasma Proteins, Sanquin Research, Amsterdam, The Netherlands; 4 Department of Experimental Vascular Medicine, Academic Medical Center, Amsterdam, the Netherlands; 5 Institute for Liver and Digestive Health, Division of Medicine, University College London (UCL), London, United Kingdom; 6 Laboratory of Catalysis and Spectroscopy, ENSICAEN, University of Caen, CNRS, 6 Boulevard du Maréchal Juin, 14050 Caen, France; 7 University of Groningen, University Medical Center Groningen, Department Bioengineering, Groningen, the Netherlands; 8 Department of Chemistry, Institute for Advanced Studies in Basic Sciences (IASBS), Iran; 9 University of Groningen, University Medical Center Groningen, Department Cell Biology, Department medical proteomics, Groningen, the Netherlands; 10 Department of Clinical Genetics, Academic Medical Center, Amsterdam, the Netherlands; 11 Department of Gastroenterology and Hepatology, Erasmus Medical Center, Rotterdam, the Netherlands; 12 Division of Cardiovascular Medicine, School of Medicine, Stanford University, Stanford, California, USA; 13 Cardiovascular Institute, School of Medicine, Stanford University, Stanford, California, USA

## Abstract

The affinity of zeolite nanoparticles (diameter of 8–12 nm) possessing high surface area and high pore volume towards human plasma proteins has been investigated. The protein composition (corona) of zeolite nanoparticles has been shown to be more dependent on the plasma protein concentrations and the type of zeolites than zeolite nanoparticles concentration. The number of proteins present in the corona of zeolite nanoparticles at 100% plasma (*in vivo* state) is less than with 10% plasma exposure. This could be due to a competition between the proteins to occupy the corona of the zeolite nanoparticles. Moreover, a high selective adsorption for apolipoprotein C-III (APOC-III) and fibrinogen on the zeolite nanoparticles at high plasma concentration (100%) was observed. While the zeolite nanoparticles exposed to low plasma concentration (10%) exhibited a high selective adsorption for immunoglobulin gamma (i.e. IGHG1, IGHG2 and IGHG4) proteins. The zeolite nanoparticles can potentially be used for selectively capture of APOC-III in order to reduce the activation of lipoprotein lipase inhibition during hypertriglyceridemia treatment. The zeolite nanoparticles can be adapted to hemophilic patients (hemophilia A (F-VIII deficient) and hemophilia B (F-IX deficient)) with a risk of bleeding, and thus might be potentially used in combination with the existing therapy.

Zeolites are low-density crystalline aluminosilicates possessing regular micropores (one-, two- and three-dimensional) with well-defined pore sizes and shapes. The well-defined structures of zeolites combine with hydrophilic/hydrophobic and porous nature render them as useful shape-selective molecular sieves and hosts for various guest molecules (organic and inorganic). A significant effort has been devoted to the preparation of zeolites with nanometer dimensions with enhanced accessibility of reactant molecules in order to achieve higher product yield/selectivity in catalytic reactions or fast diffusion in adsorption and ion exchanged processes^1^,^2^. In addition to the regular micropores, the zeolites nanoparticles contain meso- and macro-pores due to the close packing of homogeneous in size and morphology crystals. Additionally, the zeolite nanoparticles with a size smaller than 200 nm can be stabilized in suspensions with different concentrations that are colloidal stable and do not agglomerate with time^2^. Therefore, aluminosilicate and pure silicate zeolite nanoparticles are safely applied in protein adsorption for organ transplantation^3^, hemostatic material for wound healing^4^, MRI contrasting agent^5^, antibacterial agents^6^ and drug delivery^7^. Moreover, the immobilization of biomolecules on the well-developed external surface of zeolite nanoparticles is the focus of intense activity in biotechnology and biomedicine^8^,^9^,^10^.

EMT- and FAU-type zeolites have very low framework density (FD = 12.7–12.9 T/1000 Å), high porosity, diverse morphology and crystal sizes^11^. Unlike its cubic FAU polymorph that has only supercages (1.15 nm^3^), the EMT-zeolite has two cages: hypocage (0.61 nm^3^) and hypercage (1.24 nm^3^) due to different stacking of faujasite sheets. As a result, it creates different catalytic and sorption properties for both materials^11^.

Recently, it was reported that the large pore EMT-type nanosized zeolite can adsorb fibrinogen and apolipoproteins while keeping the same amount of albumin in human plasma^12^. Fibrinogen is a very complex, hydrophilic and bipolar molecule^13^,^14^,^15^,^16^,^17^,^18^. In opposite to fibrinogen, apolipoprotein C-III (APOC-III) is a simple, highly hydrophobic and non-polar molecule with a molecular weight of approximately 11 kDa. APOC-III inhibits very low-density lipoproteins (VLDL)-triglycerides, hepatic lipase (HL), and lipoprotein lipase (LPL) functions; the major function of LPL is to hydrolyze chylomicrons (CMs). As a result, the inhibition of LPL leads in the delay of degradation of triglyceride-rich particles (i.e. CM and VLDL), which is implicated in pathophysiological events such as cardiovascular diseases^19^,^20^,^21^,^22^,^23^,^24^.

The adsorption specificity of proteins has been studied for several nanoparticles including gold, super paramagnetic iron oxide, silica, and polystyrene^25^,^26^,^27^,^28^,^29^,^30^. However, to our best knowledge, the adsorption specificity of proteins on zeolite nanoparticles has not been investigated so far. As a result, it hampers the development of rational targets for biomedical applications of zeolite nanoparticles.

The aim of this paper is to study the selective sorption behavior of nanosized EMT- and FAU-zeolites for human plasma proteins with different concentrations of either the zeolites or the human plasma proteins.

## Results

### Characterization of EMT- and FAU-zeolite nanoparticles

**Figure 1:**
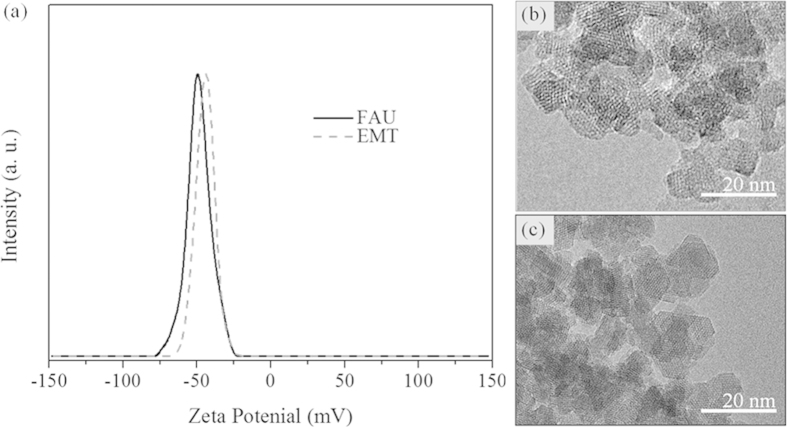
(**a**) Zeta potential curves of EMT- and FAU-zeolite suspensions, and transmission electron micrographs of (**b**) EMT- and (**c**) FAU-zeolite nanoparticles.

**Figure 2:**
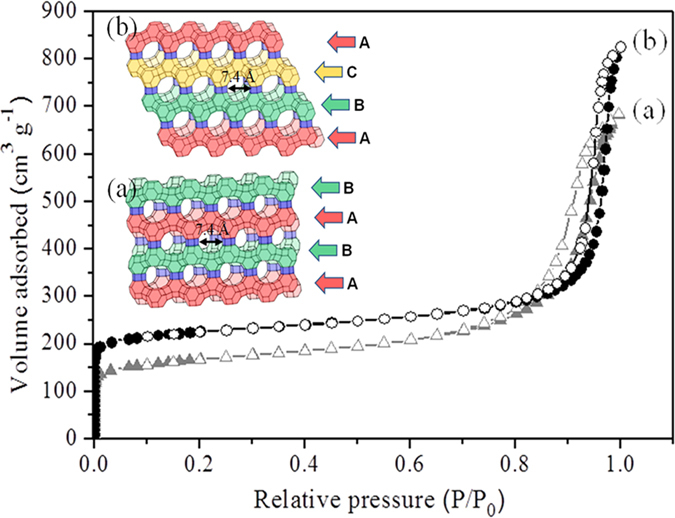
Nitrogen adsorption (closed symbol) and desorption (open symbol) isotherms of (**a**) EMT- and (**b**) FAU-zeolite nanoparticles. *Inset:* schematic presentation of the framework type of (a) EMT- and (b) FAU-zeolites, which can be described by the stacking of sodalite layers, resulting in ABABAB and ABCABC sequences, respectively.

High-resolution transmission electron microscopy (HR-TEM) and dynamic light scattering (DLS) were used to study the size and morphology of the zeolite nanocrystals. Discrete octahedral FAU- and hexagonal EMT- zeolite nanocrystals with an average diameter in the range of 8–12 nm are shown in Figure 1. Both the EMT- and FAU- zeolite nanocrystals have well developed crystalline faces. Moreover they appear as single crystals, and no crystals intergrowth was observed, which is in a good accordance with the zeta potential measurements and scanning electron microscopy study (Figure S1). The zeta potential values measured for the EMT- and FAU- zeolite nanoparticles of −44 and −50 mV, respectively, correspond to colloidal samples containing highly stable non-agglomerated crystals (Figure 1). On the other side the negative zeta potential value represent particles with negative surface charge. The surface charges of the zeolite nanoparticles are expected to play an important role in the interactions with human plasma proteins. Therefore the surface charge and charge density of both zeolite nanoparticles were determined. Both samples have almost identical surface charge density, i.e., −0.50 mC/m^2^ and −0.58 mC/m^2^ for EMT- and FAU- zeolite crystals respectively (Table 1a), while the surface charge of FAU- zeolite nanoparticles is higher (−299 mC/g) than the EMT- zeolite (−235 mC/g) (Table 1a). This can be explained with the different specific surface area of the EMT- and FAU- zeolite nanocrystals (Table 1a). The surface charge of the zeolite nanoparticles is negative, which is consistent with their high hydrophilicity. Additionally, the porosity of the zeolite nanocrystals was evaluated by nitrogen sorption measurements carried out at −196 °C (Figure 2). The N_2_ sorption isotherms of both samples resembled Type I and Type IV isotherms, indicating the presence of micropores and mesopores (textural porosity); the mesopores with an average diameter of 25 nm are originated from the close packing of the zeolite nanocrystals. The results extracted from the N_2_ sorption isotherms for EMT- and FAU- zeolite nanoparticles are summarized in Table 1. The BET specific surface area of FAU- zeolite nanoparticles is 845 m^2^/g, which is higher than for the EMT- zeolite nanoparticles (570 m^2^/g). The external surface area of EMT- and FAU-zeolite nanoparticles is 220 m^2^/g and 235 m^2^/g, respectively, which contributed of approximately 30 - 40% of their specific surface area. In addition, both samples have high total pore volume, i.e., for the EMT zeolite is 0.89 cm^3^/g and for the FAU zeolite is 1.27 cm^3^/g, which will be further beneficial for the selective sorption toward human plasma proteins.

### Semi-quantitative assessment of corona protein composition of EMT- and FAU- zeolite nanoparticles

Semi-quantitative equation is used to obtain information about the amount of each protein bound onto the surface of the EMT- and FAU- zeolite nanoparticles. All proteins identified in the experiments are described in Supporting Information (SI). The semi-quantitative assessment of each protein, namely, the normalized percentage of the spectral count for protein k (NpSpC_k_), is determined based on the total number of all peptides that were attributed to a matched protein in the nLC-MS/MS spectra (Tables 1b,c; and SI: Tables S1 and S2).

**Figure 3:**
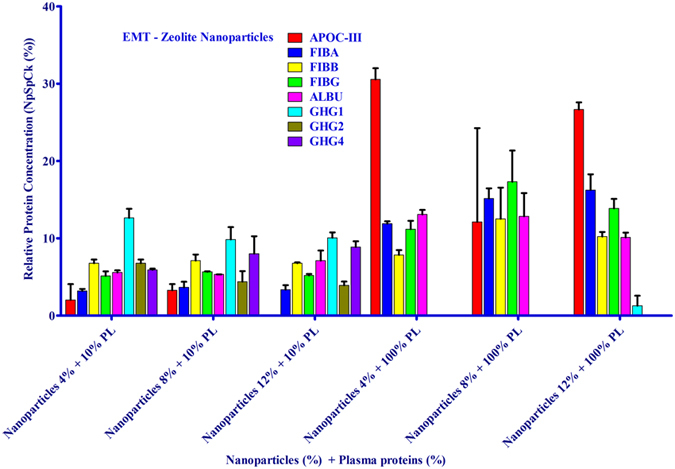
nLC-MS/MS analysis of corona-associated proteins on EMT-zeolite nanoparticles. Apolipoprotein C-III (APOC-III), fibrinogen alpha chain (FIBA), fibrinogen beta chain (FIBB), fibrinogen gamma chain (FIBG), albumin (ALBU), IGHG1, IGHG2 and IGHG4.

**Figure 4:**
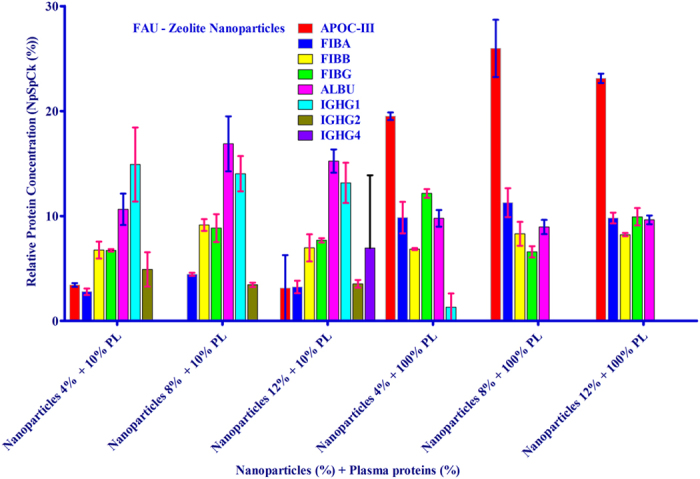
nLC-MS/MS analysis of corona-associated proteins on FAU-zeolite nanoparticles. Apolipoprotein C-III (APOC-III), fibrinogen alpha chain (FIBA), fibrinogen beta chain (FIBB), fibrinogen gamma chain (FIBG), albumin (ALBU), IGHG1, IGHG2 and IGHG4.

**Figure 5:**
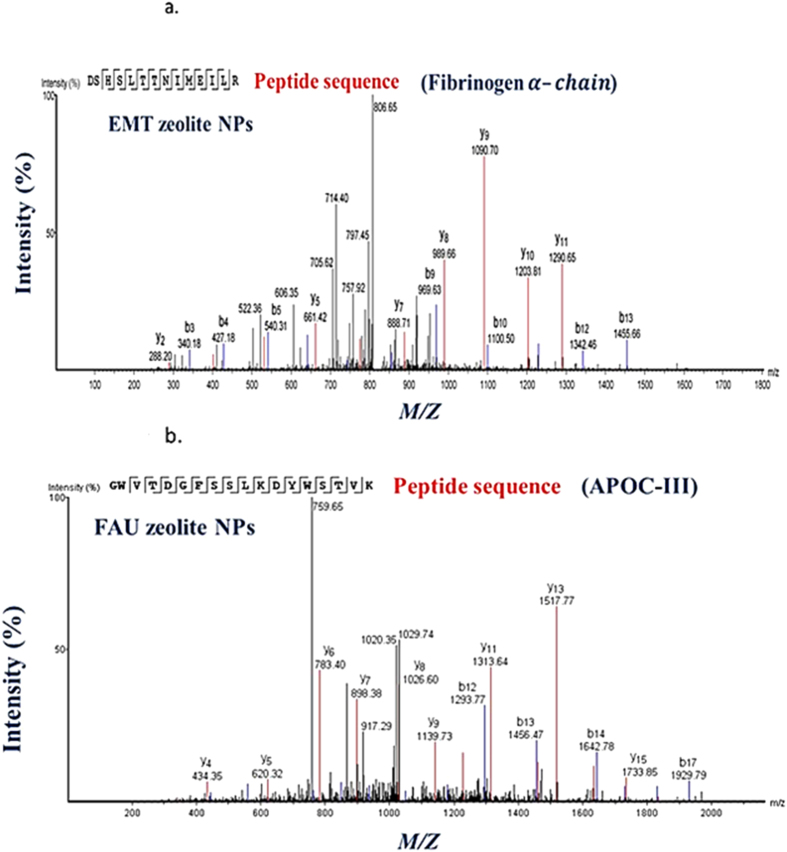
Representative nLC-MS/MS spectra of (**a**) fibrinogen α-chain after injection of extracted peptides from EMT- zeolite nanoparticles, and (**b**) APOC-III after injection of extracted peptides from FAU- zeolite nanoparticles. The peptide sequence derived from the two spectra are **GWVTDGFSSLKDYWSTVK** (APOC-III) and **DSHSLTTNIMEILR** (fibrinogen α-chain), respectively.

The affinity of the EMT- and FAU- zeolite nanoparticles towards human plasma proteins was investigated by incubating zeolite nanoparticles with plasma proteins using one of the following strategies: (1) the concentration of nanoparticles was kept constant but the plasma concentration was varied, and (2) the plasma concentration was kept constant but the concentration of zeolite nanoparticles was varied. The identification of proteins present on the surface of EMT- and FAU-zeolite nanoparticles (SI: Tables S1 and S2) was carried out using nLC-MS/MS (LTQ-ORBITRAP-XL; tandem mass spectrometry) combined with PEAKS DB (software) analysis. The nLC-MS/MS results show that with increasing the plasma concentrations from 10% to 100%, the APOC-III and the three chains of fibrinogen (FIBA, FIBB and FIBG) are bound on the surface of the zeolite nanoparticles. The amount of adsorption was less dependent on the concentration of zeolite nanoparticles than plasma concentrations used (Figures 3 and 4; and SI: Tables S1 and S2). After exposure of zeolite nanoparticles to very low plasma concentrations (10%), the zeolites exhibit a high affinity for immunoglobulin gamma (i.e. IGHG1, IGHG2 and IGHG4) as compared to the high plasma concentrations (Figures 3 and 4). Notably, upon using EMT-zeolite nanoparticles with 100% plasma, the APOC-III showed an adsorption of 30%. Furthermore, all three chains of fibrinogen (FIBA, FIBB, and FIBG) demonstrated a high affinity toward EMT-zeolite nanoparticles even at low concentrations (4–12%) incubated with 10% plasma. As a representative example, the spectra of Fibrinogen α-chain and APOC-III after injection of the extracted peptides from EMT and FAU zeolite nanoparticles are depicted in Figure 5 (a,b).

### Interaction of APOC-III and fibrinogen with EMT- and FAU-zeolite nanoparticles

**Figure 6:**
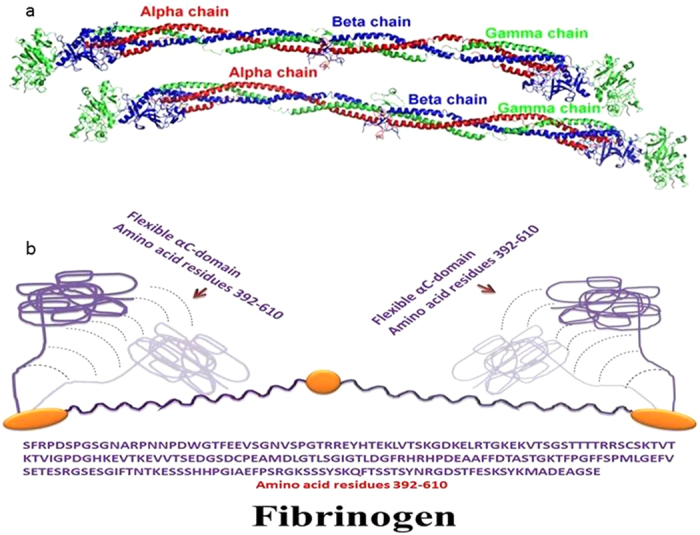
(**a**) Crystal structure of fibrinogen (crystal structure was used from PDB entry 3GHG), (**b**) schematic diagram of fibrinogen structure showing the flexibility of positively charged αC-domain. The amino acid sequence of αC-domain, amino acid residues 392–610, is shown below the diagram.

The APOC-III is adsorbed on the surface of both EMT- and FAU-zeolite nanoparticles (Figure S2). The positively charged amino acid residues of APOC-III and the negatively charged EMT- and FAU-zeolite nanoparticles facilitate the electrostatic interactions and thus the adsorption of APOC-III on the zeolite surfaces. Fibrinogen is a bipolar molecule with negatively charged E and D domains, and positively charged αC-domain (PDB entry: 3GHG; Figure 6a). The αC-domain of fibrinogen Aα-chain (starts from amino acid 392 to 610) is highly flexible, mobile and positively charged^31^,^32^,^33^,^34^. This region shows positive electrostatic character, which is potentially responsible for the interaction between the fibrinogen and the negatively charged EMT- and FAU-zeolite nanoparticles. A schematic diagram of human fibrinogen structure and a flexible αC-domain of the fibrinogen are depicted in Figure 6b.

### Effect of EMT- and FAU-zeolite nanoparticles on blood coagulation

**Figure 7:**
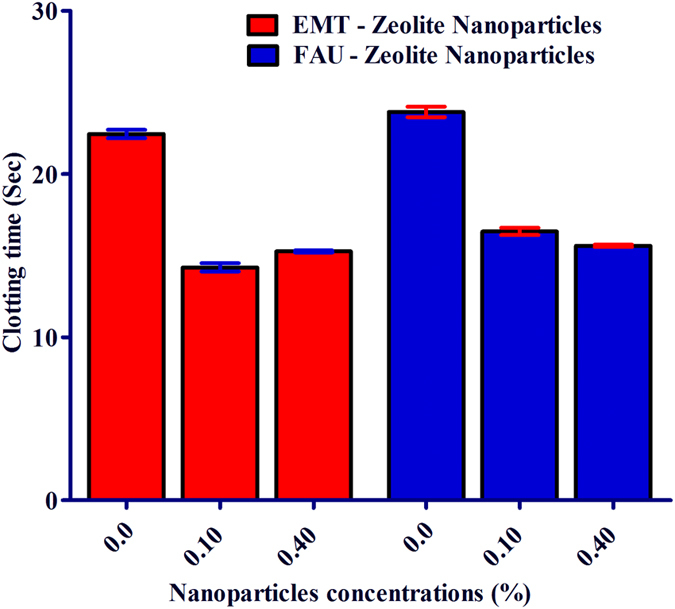
The effect of EMT- and FAU-zeolite nanoparticles on clotting time: prothrombin test (PT) is performed in pooled plasma with and without EMT- and FAU-zeolite nanoparticles (incubation for 30 min) in KC-10 coagulometer. Clotting time is expressed in seconds (Sec). Zeolite nanoparticles (0.1% and 0.4%) are incubated in 100 μl of 100% pooled plasma.

Fibrinogen is a glycoprotein that helps in the formation in blood clots. Since the fibrinogen has strong tendency to bind to both zeolite nanoparticles, thus it is very important to investigate their effect on clotting time. In respect to this, coagulometer was applied to determine prothrombin time (PT), i.e., a test that measures how long it takes the blood to clot. Without adding zeolite nanoparticles, the thrombin time is 23 sec (Figure 7). Upon treatment of 50% plasma for 30 min with 0.1% EMT- and FAU-zeolite nanoparticles, the clotting time is shortened with 5–7 seconds. One of the possibilities could be potentially due to the adsorption of fibrinogen to zeolite nanoparticles in the pooled plasma and hence, enhancing the rate of clot formation. The FAU-zeolite nanoparticles with higher surface area, higher total pore volume and higher surface charge density show clotting time of 18 sec, while for the EMT- zeolite nanoparticles is 16 sec. This indicates that EMT-zeolite nanoparticles with higher hydrophilicity (Si/Al ratio of 1.14) are more selectively in interacting with the fibrinogens and leads to enrichment of fibrinogens in blood, which is essential in blood clotting. Thus, these NPs accelerate the time of blood clot formation and the mechanism whereby these NPs accelerate coagulation remains to be elucidated.

## Discussion

Nanosized EMT- and FAU-zeolites open the possibility to use them in new biological and clinical applications (biological fluid)^35^,^36^,^37^. Of note, proteins are one of the most important components of biological fluid such as plasma system (sub-fraction of blood) and the interactions with zeolite nanoparticles is important to be studied prior further use.

There are ignorable differences in the number of identified proteins bound on the surface of the EMT- and FAU-zeolite nanoparticles (at 4% NPs) when they were exposed to 10% plasma proteins, and this could be due to (1) the low abundant proteins present in diluted plasma, and (2) no real competition between proteins during the adsorption on zeolite nanoparticles with large external surface. The latter phenomenon could be mediated by the presence of a few very high-abundance proteins albumin and immunoglobulin G (IgG)^38^,^39^,^40^, which are responsible for the constitution of 70% and 20% of plasma proteins, respectively. This is the reason why the two major proteins in the EMT- and FAU-corona (protein layer of nanoparticles) exposed to 10% plasma only are albumin and IgG. It should be noted that the two major functions of albumin are binding and transporting the materials^41^. Importantly, the number of proteins present in the corona of zeolite nanoparticles is much less at 100% plasma exposure (*in vivo* state) than with 10% plasma. Therefore the assumption that there is a competition between proteins to occupy the corona of the zeolite nanoparticles is reasonable. Thus, the protein composition of corona in EMT- and FAU-zeolite nanoparticles appeared to be more dependent on plasma concentration and the type of zeolite (e.g. may be the external surface area of zeolite crystals) than NPs concentrations with respect to APOC-III, IGHG1, IGHG2 and IGHG3. Since EMT-zeolite nanoparticles are more hydrophilic than FAU-zeolite nanoparticles, it is possible to assume that the high adsorption on EMT- zeolite may be due to the hydrophilicity.

### Selective adsorption of APOC-III and fibrinogen on the surface of EMT- and FAU-zeolite nanoparticles

The selective adsorption of APOC-III on the surface of EMT- and FAU-zeolite nanoparticles is most likely *via* electrostatic interactions between the solvent exposed positively charged amino acid residues of APOC-III and the negatively charged EMT- and FAU-zeolites. Specifically, there are two regions in the structures of APOC-III, that the side chains of amino acid residues are solvent exposed, and thus they can electrostatically interact with the negatively charged zeolite nanocrystals (Figure S2). The region including amino acid residues *Lys17*, *His18*, *Lys21* and *Lys24*, and the area containing the solvent exposed amino acid residues *Lys51, Lys58* and *Lys60* are highly effective in binding to the negatively charged EMT- and FAU-zeolite nanoparticles^42^. Furthermore, APOC-III is an inhibitor of LPL activation and it is considered as a risk factor for cardiovascular diseases. The two types zeolites can potentially be used for selectively capture of APOC-III, and to reduce the activation of lipoprotein lipase inhibition in hypertriglyceridemia treatment.

The application of *in vivo* plasma proteins (i.e. 100%) appeared to be essential for a strong binding of fibrinogen (all three chains) to the EMT- and FAU-zeolite nanoparticles. Fibrinogen D and E domains are rich in aspartic acid (*Asp*) and glutamic acid (*Glu*) residues, and these sections are negatively charged. In contrast, the αC-domain (remaining part of carboxyl terminal of both alpha chains, which are not present in D domain) is positively charged because this region is rich in arginine (*Arg*) and lysine (*Lys*). The fibrinogen is a dipolar molecule and it can be one of the potential reasons that facilitate the adsorption of fibrinogen on the surface of negatively charged EMT- and FAU-zeolite nanoparticles. Importantly, a maximum concentration of fibrinogen is a guarantee for its strong adsorption to both zeolite nanoparticles. Since these two nanoparticles are negatively charged, it is possible that fibrinogen is linked to the surface of the zeolite nanoparticles *via* αC-domains, which are positively charged. Other possibility is that the fibrinogen molecules, which are dipolar (D and E domains are negatively charged and αC-domain positively charged) and very stable, and hold tightly together. Most likely, the positive charged fibrinogen domain binds to the zeolite nanoparticles.

### Effect of EMT- and FAU-zeolite nanoparticles on coagulation

It is very important to shed light on the role of the zeolite nanoparticles in biological functions such as coagulation system, which play an important role in wound healing process. Since the EMT- and FAU-zeolite nanoparticles show a very high selectivity for fibrinogen, it is necessary to measure the effect of these zeolite nanoparticles on fibrin formation. Notably, both zeolite nanoparticles accelerate the fibrin forming process. This property of the EMT- and FAU-zeolite nanoparticles can be adapted to hemophilic patients (hemophilia A (F-VIII deficient) and hemophilia B (F-IX deficient)) with a risk of bleeding. The present therapy of such patients is based on treatment with F-VIII extracts. Thus, the EMT- and FAU-zeolite nanoparticles might be potentially used as a complementary therapy in combination with the existing one.

## Conclusions

This study provides an evidence for high specific adsorption of APOC-III and fibrinogen on EMT- and FAU-zeolite nanoparticles. There are three factors, that determine the protein content of corona; 1- plasma concentrations, 2- the type of zeolite NPs (external surface area and hydrophilicity of zeolite nanocrystals), and 3- NPs concentrations (but less effect). Moreover, it was found that these NPs accelerate the time of blood clot formation, which can be adapted to hemophilic patients (hemophilia A (F-VIII deficient) and hemophilia B (F-IX deficient)) with a risk of bleeding, and thus might be potentially used in combination with the existing therapy. Also, the zeolite nanoparticles can potentially be used for selectively capture of APOC-III in order to reduce the activation of lipoprotein lipase inhibition during hypertriglyceridemia treatment.

## Experimental section

### Synthesis and characterization of EMT- and FAU-zeolite nanoparticles

The EMT-zeolite nanoparticles with a diameter of 8–12 nm were synthesized from a clear precursor suspension with a molar composition of 18.45Na_2_O: 5.15SiO_2_: 1Al_2_O_3_: 240.3H_2_O^35^. This suspension was subjected to hydrothermal synthesis at 30 °C for 36 h. The crystalline EMT-zeolite nanoparticles were then purified by a high-speed centrifugation (75465 × g for 2 h) and re-dispersed in double distilled water; this procedure was repeated six times until the final colloidal suspension has a pH of 7.0; the solid concentration of EMT-zeolite nanoparticles was adjusted to 5 wt%.

The FAU-zeolite nanoparticles with a diameter of 8–12 nm were synthesized from a clear precursor suspension with a molar composition: 9Na_2_O: 10SiO_2_: 0.7Al_2_O_3_: 160H_2_O^43^. The suspension was subjected to hydrothermal synthesis at 50 °C for 45 h. The FAU-zeolite nanoparticles were then purified by a high-speed centrifugation (75465 × g for 2 h) and re-dispersed in double distilled water; this procedure was repeated three times until the final colloidal suspensions have a pH of 7.0; the solid concentration of FAU-nanoparticles was then adjusted to 5 wt%.

The chemical compositions of zeolite nanocrystals were determined by X-ray fluorescence (XRF) spectroscopy using a MagiX PHILIPS PW2540FEI. Transmission electron microscope (TEM) and scanning electron microscope (SEM) operating at 300 kV and 30 kV using a JEOL and a Philips XL-30, respectively were applied to study the crystalline EMT- and FAU-zeolite nanoparticles. The hydrodynamic diameters and zeta potential of the zeolite nanoparticles in the suspensions were determined with a Malvern Zetasizer Nano. The samples were first re-suspended in 10 mL of 1.0 mM KCl and the zeta potential of the particles was recorded. The surface charge density, σ, is calculated from the Grahame equation^44^:


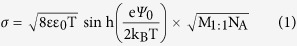


where, εε_0_ is the dielectric permittivity of zeolite (FAU = 1.3458 × 10^−11^ AsV^−1^ m^−1^, EMT = 1.3547 × 10^−11^ AsV^−1^ m^−1^), k_B_ is the Boltzmann constant (1.381 × 10^−23^ JK^−1^), ψ_0_ is the surface potential or zeta potential of the zeolite suspensions in 1.0 mM KCl, e is the electronic charge (1.602 × 10^−19^ C), T is the absolute temperature (298 K), N_A_ is the Avogadro’s number (6.022 × 10^23^ mol^−1^) and M_1:1_ is the concentration of KCl (1.0 mM). The surface charge of zeolite nanoparticles, Q, was calculated using the following equation:


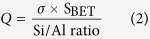


where, σ is the surface charge density, S_BET_ is the specific surface area, and Si/Al ratio is the silicon to aluminum ratio of the zeolite nanoparticles.

The porosity of zeolite nanoparticles were measured *via* adsorption and desorption of nitrogen using micrometrics ASAP 2010 volumetric adsorption analyzer. Samples were degassed at 250 °C under vacuum overnight prior to the measurement. The external surface area and micropore volume were estimated by alpha-plot method using Silica-1000 (22.1 m^2^ g^−1^) as a reference.

### Collection of plasma

Informed consent was obtained from all subjects and this was approved by the Medical Ethical Committee of the Academic Medical Center Amsterdam under number W11_084/#11.17.864. The methods were carried out in accordance with the approved international guidelines. Blood was collected from healthy volunteers in tubes containing sodium citrate (Na_3_C_6_H_5_O_7_, Becton Dickson & Co., The Netherlands) as anticoagulant. Thereafter, the blood was centrifuged at 1500 × g for 20 min at 4 °C to collect plasma and subsequently all plasma samples were pooled and stored at −80 °C for further analysis.

### Semi-quantitative assessment of each protein presents on corona zeolite nanoparticles

A semi-quantitative assessment of the protein amounts was conducted through the application of the spectral counting (SpC), based on the following equation^12^,^25^:


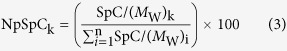


where NpSpC_k_ is the normalized percentage of the spectral count for protein k, SpC is the spectral count identified, and M_w_ is the molecular weight (kDa) of protein k. The SpC of each identified protein was normalized to the protein mass and expressed as the relative quantity of protein. This correction is based on the protein size and evaluates the real contribution of each protein present in the corona of zeolite nanoparticles.

In order to identify the proteins with high affinity towards EMT- and FAU-zeolite nanoparticles, different concentrations of zeolite nanoparticles (4 and 12 wt%) were incubated for 60 min with different human plasma concentrations (10 and 100%). For comparison, two blank samples were applied: (1) Milli-Q water containing 1% formic acid, and (2) 100 μL nanoparticles were incubated with Milli-Q water containing 1% formic acid. After the incubations, all samples were centrifuged three times (24000 × g for 30 min) to remove the free plasma. Subsequently, all samples were subjected to tryptic digestion (trypsin: 100 ng/sample) at 37 °C for 24 h. Then the samples were centrifuged at 24000 × g for 60 min. The peptides were collected, and the zeolite nanoparticles were again re-suspended in 20 μL Milli-Q water containing 2% formic acid (three times vortexed during 45 min incubation and centrifuged at 24000 × g for 30 min). Finally, the peptides were collected and added to the first elution. All samples were then reduced with 1% dithiothreitol to break disulfide bridges. Finally, 2 μL of each sample-derived peptides fraction was injected into nLC-MS/MS (LTQ-ORBITRAP-XL). For each sample, two or three experiments were performed, measured by nLC-MS/MS to ensure high accuracy in the analysis.

The peptides were analyzed by nLC-MS/MS on an Ultimate 3000 system (Dionex, Amsterdam, The Netherlands) interfaced online with a LTQ Orbitrap XL mass spectrometer (Thermo Fisher Scientific, San Jose, CA). Re-dissolved peptides were loaded onto a trapping microcolumn (5 mm × 300 μm i.d.) packed with C18 PepMAP100 particles (5 μm, Dionex) in 0.1% formic acid at a flow rate of 20 μL min^−1^. Upon loading and washing, the peptides were back flush eluted onto a nano-column (15 cm × 75 μm i.d.) packed with C18 PepMAP100 particles (3 μm, Dionex). The following mobile phase gradient was executed at a flow rate of 300 nL min^−1^: 5–50% of solvent B in 93 min; 50–80% B in 5 min; 80% B for 10 min, and back to 5% B in 5 min. Solvent A was 100:0 H_2_O–acetonitrile (v/v) with 0.1% formic acid, and solvent B was 10:90 H_2_O–acetonitrile (v/v) with 0.1% formic acid. Peptides were infused in the mass spectrometer *via* a dynamic nano-spray probe (Thermo Electron Corp.) with a stainless steel emitter (Proxeon, Odense, DK). The typical spray voltage was 1.6 kV with no sheath and auxiliary gas flow; the ion transfer tube temperature was 200 °C. The mass spectrometer was operated in data-dependent mode. Automated gain control (AGC) was set to 5 × 10^5^ charges and 1 × 10^4^ charges for MS/MS at the linear ion trap analyzer. The Data Dependent Acquisition (DDA) cycle consisted of the survey scan within m/z 300–1300 at the orbitrap analyzer with target mass resolution of 60000 (full width half maximum at m/z 400) followed by MS/MS fragmentation of the five most intense precursor ions under the relative collision energy of 35% in the linear trap. Singly charged ions were excluded from MS/MS experiments, and m/z of fragmented precursor ions were dynamically excluded for 90 s. The ion selection threshold for triggering MS/MS experiments was set to 500 counts. An activation parameter, q, of 0.25 and an activation time of 30 ms were applied.

PEAKS DB^45^ (version 6.1) was applied to analyze the spectra/sample generated by nLC-MS/MS to identify proteins that bind to the layer (corona) of EMT- and FAU-zeolite nanoparticles. The total spectra per protein were determined by PEAKS DB to calculate the NpSpC_k_ values. The structure of APOC-III and fibrinogen were drawn with PyMOL^46^.

### Effect of EMT- and FAU-zeolite on coagulation: prothrombin time (PT)

To measure the time that is needed to clot the plasma portion of blood, prothrombin test (PT) was performed in pooled plasma with and without EMT- and FAU-zeolite nanoparticles in a KC-10 coagulometer. Three concentrations of EMT- and FAU-zeolite nanoparticles (0, 0.1 and 0.4%) were incubated with 100% pooled plasma derived from healthy volunteers for 30 min at room temperature and vortexed very gentle intermittently. Thereafter, prothrombin reagent was 10 times diluted; it contains a tissue factor and Ca^2+^ providing calcium ions supplement in the solution kept at 37 °C. From this reagent, 100 μL was added to each sample to start the reaction. The time between the addition of the thrombin and the clot formation is recorded as the thrombin clotting time (Sec).

## Additional Information

**How to cite this article**: Rahimi, M. *et al.* Zeolite Nanoparticles for Selective Sorption of Plasma Proteins. *Sci. Rep.* **5**, 17259; doi: 10.1038/srep17259 (2015).

## Supplementary Material

Supplementary Data

## Figures and Tables

**Table 1:**
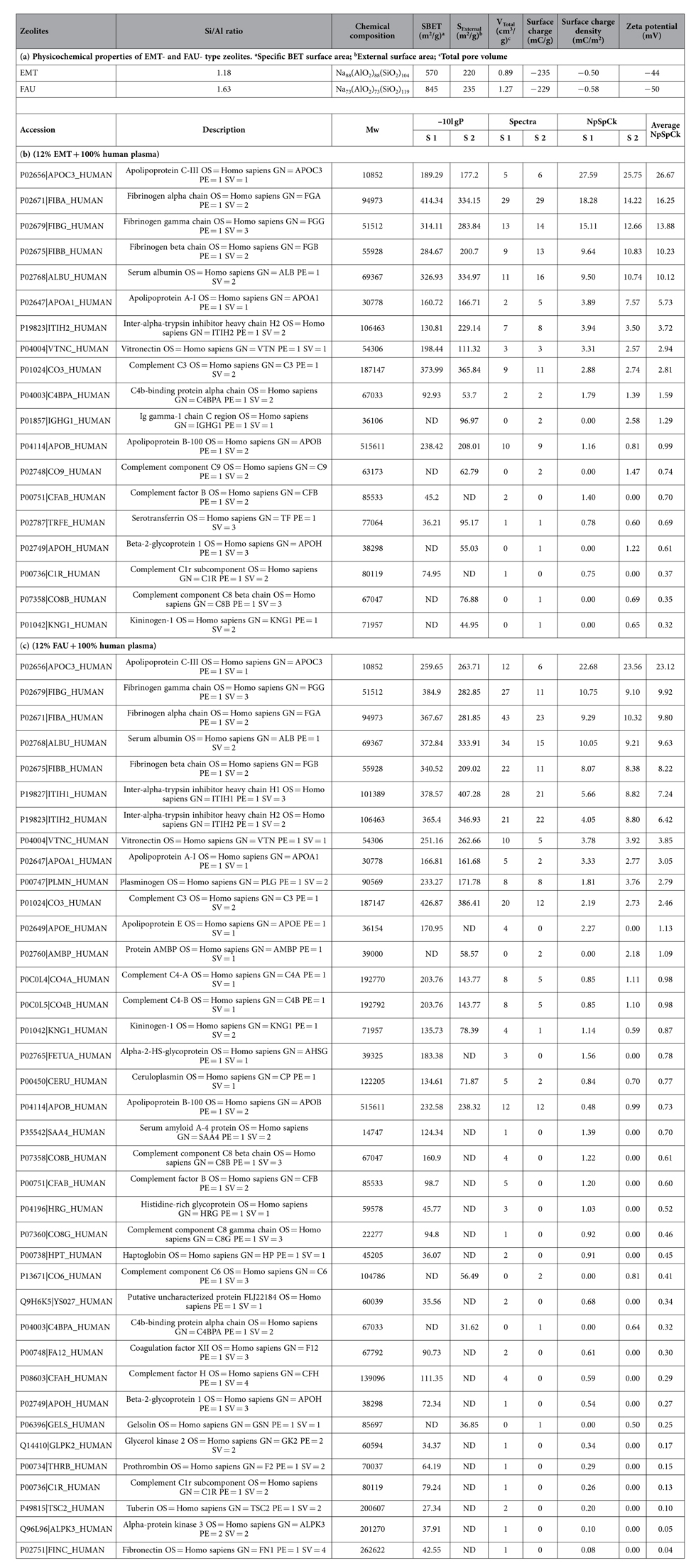
NLC-MS/MS combined with PEAKS DB analysis of the corona (protein content) and physicochemical properties of zeolite nanoparticles (NPs).

Table 1a shows physicochemical surface properties of EMT and FAU zeolite NPs. Table 1b represents EMT zeolite NPs (12% EMT + 100% human plasma) and Table 1c is FAU zeolite NPs (12% FAU + 100% human plasma). The accession number, gene name, species (Human), protein description, identification score (-10lgP), molecular weight (Mw) in kDa, total spectra per protein of EMT (12%) and FAU (12%) zeolite NPs incubated with human plasma (100%), together with their relative amount (NpSpCk value). Sample (S). All protein coverage of different combination (EMT (%) and plasma (%), and (FAU (%) and plasma (%)) were reported in Supplementary Information.
