# Supplementary material for: Zeolite Nanoparticles for Selective Sorption of Plasma Proteins
Source: Sci Rep. 2015 Nov 30;5:17259. doi: 10.1038/srep17259 (PMC4663482; doi:10.1038/srep17259)
Supplement: Supplementary Data [file srep17259-s1.pdf]

Supplementary Data

Zeolite Nanoparticles for Selective Sorption of Plasma Proteins

M. Rahimi[1#,] E.-P. Ng[2#,], K. Bakhtiari[3,4], M. Vinciguerra5, H. Ali Ahmad[6], H. Awala[6], S. Mintova[6], M. Daghighi[7], F. Bakhshandeh[8], M. de Vries[9], M. M. Motazacker[10], M. P. Peppelenbosch[11!] , M. Mahmoudi[12,13!,], F. Rezaee[9,11,*]

[1]University of Groningen, University Medical Center Groningen, Department of Medical Biology, Groningen, the Netherlands.
[2]School of Chemical Sciences, University Sains Malaysia, 11800 USM, Malaysia
[3]Department of Plasma Proteins, Sanquin Research, Amsterdam, The Netherlands
[4]Department of Experimental Vascular Medicine, Academic Medical Center, Amsterdam, the Netherlands
[5]Institute for Liver and Digestive Health, Division of Medicine, University College London (UCL), London, United Kingdom.
[6]Laboratory of Catalysis and Spectroscopy, ENSICAEN, University of Caen, CNRS, 6 Boulevard du Maréchal Juin, 14050 Caen, France.
[7]University of Groningen, University Medical Center Groningen, Department Bioengineering, Groningen, the Netherlands.
[8]Department of Chemistry, Institute for Advanced Studies in Basic Sciences (IASBS), Iran
[9]University of Groningen, University Medical Center Groningen, Department Cell Biology, Department medical proteomics, Groningen, the Netherlands
[10]Department of Clinical Genetics, Academic Medical Center, Amsterdam, the Netherlands
[11]Department of Gastroenterology and Hepatology, Erasmus Medical Center, Rotterdam, the Netherlands
[12]Division of Cardiovascular Medicine, School of Medicine, Stanford University, Stanford, California, USA
[13]Cardiovascular Institute, School of Medicine, Stanford University, Stanford, California, USA

#Equally contributed
!Equally contributed

*Corresponding author: Email: (FR) f.rezaee@erasmusmc.nl , f.rezaee@med.umcg.nl,

Table S1 (a- f): nLC-MS/MS combined with PEAKS DB analysis of the corona (protein content) of Nano-sized EMT zeolite NPs. The accession number, gene name, species (Human), protein description, identification score (-10lgP), molecular wight (Mw) in kDa, total spectra per protein of EMT (4%, 8%, and 12%) zeolite NPs incubated with human plasma (10%,50%, and 100%), together with their relative amount (NpSpCk value).Sample (S).

Table S1a: (4% EMT + 10% human plasma)

Accession	Description	Mw	-10lgP S 1	-10lgP S 2	Spectra S 1	Spectra S 2	NpSpCk S 1	NpSpCk S 2	Average NpSpCk
**P01857|IGHG1_HUMAN**	Ig gamma-1 chain C region OS=Homo sapiens GN=IGHG1 PE=1 SV=1	36106	305.32	319.72	28	32	11.45	13.83	12.64
**P01861|IGHG4_HUMAN**	Ig gamma-4 chain C region OS=Homo sapiens GN=IGHG4 PE=1 SV=1	35941	314.59	323.72	25	27	10.27	11.72	10.99
**P02675|FIBB_HUMAN**	Fibrinogen beta chain OS=Homo sapiens GN=FGB PE=1 SV=2	55928	286.85	281.91	24	26	6.33	7.25	6.79
**P01859|IGHG2_HUMAN**	Ig gamma-2 chain C region OS=Homo sapiens GN=IGHG2 PE=1 SV=2	35901	247.19	254.22	14	14	5.76	6.08	5.92
**P02768|ALBU_HUMAN**	Serum albumin OS=Homo sapiens GN=ALB PE=1 SV=2	69367	320.81	330.38	25	26	5.32	5.85	5.58
**P02679|FIBG_HUMAN**	Fibrinogen gamma chain OS=Homo sapiens GN=FGG PE=1 SV=3	51512	305.08	265.61	20	15	5.73	4.54	5.14
**P04196|HRG_HUMAN**	Histidine-rich glycoprotein OS=Homo sapiens GN=HRG PE=1 SV=1	59578	270.99	227.92	17	13	4.21	3.40	3.81
**P02647|APOA1_HUMAN**	Apolipoprotein A-I OS=Homo sapiens GN=APOA1 PE=1 SV=1	30778	223.08	239.97	6	8	2.88	4.06	3.47
**P02671|FIBA_HUMAN**	Fibrinogen alpha chain OS=Homo sapiens GN=FGA PE=1 SV=2	94973	302	282.67	19	21	2.95	3.45	3.20
**P01024|CO3_HUMAN**	Complement C3 OS=Homo sapiens GN=C3 PE=1 SV=2	187147	427.91	427.23	36	40	2.84	3.33	3.09
**P02655|APOC2_HUMAN**	Apolipoprotein C-II OS=Homo sapiens GN=APOC2 PE=1 SV=1	11284	126.19	134.68	2	2	2.62	2.77	2.69
**P01834|IGKC_HUMAN**	Ig kappa chain C region OS=Homo sapiens GN=IGKC PE=1 SV=1	11609	146.13	86.03	2	2	2.54	2.69	2.62
**P04114|APOB_HUMAN**	Apolipoprotein B-100 OS=Homo sapiens GN=APOB PE=1 SV=2	515611	478.69	458.45	76	76	2.18	2.30	2.24
**P02656|APOC3_HUMAN**	Apolipoprotein C-III OS=Homo sapiens GN=APOC3 PE=1 SV=1	10852	138.04	ND	3	0	4.08	0.00	2.04
**P36955|PEDF_HUMAN**	Pigment epithelium-derived factor OS=Homo sapiens GN=SERPINF1 PE=1 SV=4	46312	161.59	153.82	6	5	1.91	1.68	1.80
**P0C0L4|CO4A_HUMAN**	Complement C4-A OS=Homo sapiens GN=C4A PE=1 SV=1	192770	287.29	300.24	17	21	1.30	1.70	1.50
**P0C0L5|CO4B_HUMAN**	Complement C4-B OS=Homo sapiens GN=C4B PE=1 SV=1	192792	287.29	300.24	17	21	1.30	1.70	1.50
**P0CG06|LAC3_HUMAN**	Ig lambda-3 chain C regions OS=Homo sapiens GN=IGLC3 PE=1 SV=1	11237	94.25	108.57	1	1	1.31	1.39	1.35
**P0CF74|LAC6_HUMAN**	Ig lambda-6 chain C region OS=Homo sapiens GN=IGLC6 PE=4 SV=1	11277	94.25	108.57	1	1	1.31	1.38	1.35
**P0CG05|LAC2_HUMAN**	Ig lambda-2 chain C regions OS=Homo sapiens GN=IGLC2 PE=1 SV=1	11294	94.25	108.57	1	1	1.31	1.38	1.34

**A0M8Q6|LAC7_HUMAN**	Ig lambda-7 chain C region OS=Homo sapiens GN=IGLC7 PE=1 SV=2	11303	94.25	108.57	1	1	1.31	1.38	1.34
**P0CG04|LAC1_HUMAN**	Ig lambda-1 chain C regions OS=Homo sapiens GN=IGLC1 PE=1 SV=1	11348	94.25	108.57	1	1	1.30	1.37	1.34
**P01625|KV402_HUMAN**	Ig kappa chain V-IV region Len OS=Homo sapiens PE=1 SV=2	12640	110.79	85.98	1	1	1.17	1.23	1.20
**P00751|CFAB_HUMAN**	Complement factor B OS=Homo sapiens GN=CFB PE=1 SV=2	85533	199.46	153.09	6	7	1.04	1.28	1.16
**P06312|KV401_HUMAN**	Ig kappa chain V-IV region (Fragment) OS=Homo sapiens GN=IGKV4-1 PE=4 SV=1	13380	110.79	85.98	1	1	1.10	1.17	1.13
**P01031|CO5_HUMAN**	Complement C5 OS=Homo sapiens GN=C5 PE=1 SV=4	188304	252.48	258.34	15	13	1.18	1.08	1.13
**P06313|KV403_HUMAN**	Ig kappa chain V-IV region JI OS=Homo sapiens PE=4 SV=1	14633	110.79	85.98	1	1	1.01	1.07	1.04
**P06314|KV404_HUMAN**	Ig kappa chain V-IV region B17 OS=Homo sapiens PE=2 SV=1	14966	110.79	85.98	1	1	0.99	1.04	1.01
**P00747|PLMN_HUMAN**	Plasminogen OS=Homo sapiens GN=PLG PE=1 SV=2	90569	165.56	165.1	5	5	0.81	0.86	0.84
**P02652|APOA2_HUMAN**	Apolipoprotein A-II OS=Homo sapiens GN=APOA2 PE=1 SV=1	11175	55.8	ND	1	0	1.32	0.00	0.66
**B9A064|IGLL5_HUMAN**	Immunoglobulin lambda-like polypeptide 5 OS=Homo sapiens GN=IGLL5 PE=2 SV=2	23063	94.25	108.57	1	1	0.64	0.68	0.66
**P02745|C1QA_HUMAN**	Complement C1q subcomponent subunit A OS=Homo sapiens GN=C1QA PE=1 SV=2	26017	127	ND	2	0	1.13	0.00	0.57
**P19823|ITIH2_HUMAN**	Inter-alpha-trypsin inhibitor heavy chain H2 OS=Homo sapiens GN=ITIH2 PE=1 SV=2	106463	140.44	134.86	4	4	0.55	0.59	0.57
**P04004|VTNC_HUMAN**	Vitronectin OS=Homo sapiens GN=VTN PE=1 SV=1	54306	145.23	142.8	2	2	0.54	0.57	0.56
**P02751|FINC_HUMAN**	Fibronectin OS=Homo sapiens GN=FN1 PE=1 SV=4	262622	242.97	271.74	8	9	0.45	0.53	0.49
**P02766|TTHY_HUMAN**	Transthyretin OS=Homo sapiens GN=TTR PE=1 SV=1	15887	91.37	ND	1	0	0.93	0.00	0.46
**P00736|C1R_HUMAN**	Complement C1r subcomponent OS=Homo sapiens GN=C1R PE=1 SV=2	80119	88.75	91.34	2	3	0.37	0.58	0.48
**P07358|CO8B_HUMAN**	Complement component C8 beta chain OS=Homo sapiens GN=C8B PE=1 SV=3	67047	78.34	131.48	2	2	0.44	0.47	0.45
**P08603|CFAH_HUMAN**	Complement factor H OS=Homo sapiens GN=CFH PE=1 SV=4	139096	166.03	124.98	4	4	0.42	0.45	0.44
**P02649|APOE_HUMAN**	Apolipoprotein E OS=Homo sapiens GN=APOE PE=1 SV=1	36154	ND	118.05	0	2	0.00	0.86	0.43
**P01877|IGHA2_HUMAN**	Ig alpha-2 chain C region OS=Homo sapiens GN=IGHA2 PE=1 SV=3	36526	36.1	ND	2	0	0.81	0.00	0.40
**P01876|IGHA1_HUMAN**	Ig alpha-1 chain C region OS=Homo sapiens GN=IGHA1 PE=1 SV=2	37655	36.1	ND	2	0	0.78	0.00	0.39
**P02760|AMBP_HUMAN**	Protein AMBP OS=Homo sapiens GN=AMBP PE=1 SV=1	39000	69.18	90.2	1	1	0.38	0.40	0.39
**P13671|CO6_HUMAN**	Complement component C6 OS=Homo sapiens GN=C6 PE=1 SV=3	104786	107.14	119.3	2	3	0.28	0.45	0.36
**O95445|APOM_HUMAN**	Apolipoprotein M OS=Homo sapiens GN=APOM PE=1 SV=2	21253	ND	80.52	0	1	0.00	0.73	0.37
**P04003|C4BPA_HUMAN**	C4b-binding protein alpha chain OS=Homo sapiens GN=C4BPA PE=1 SV=2	67033	117.73	61.19	2	1	0.44	0.23	0.34
**P01042|KNG1_HUMAN**	Kininogen-1 OS=Homo sapiens GN=KNG1 PE=1 SV=2	71957	ND	64.06	0	3	0.00	0.65	0.33
**P10909|CLUS_HUMAN**	Clusterin OS=Homo sapiens GN=CLU PE=1 SV=1	52495	69.78	79.12	1	1	0.28	0.30	0.29

**Q96PD5|PGRP2_HUMAN**	N-acetylmuramoyl-L-alanine amidase OS=Homo sapiens GN=PGLYRP2 PE=1 SV=1	62217	59.02	58.72	1	1	0.24	0.25	0.24
**P07357|CO8A_HUMAN**	Complement component C8 alpha chain OS=Homo sapiens GN=C8A PE=1 SV=2	65163	ND	111.78	0	2	0.00	0.48	0.24
**P03951|FA11_HUMAN**	Coagulation factor XI OS=Homo sapiens GN=F11 PE=1 SV=1	70109	69.62	74.38	1	1	0.21	0.22	0.22
**P02749|APOH_HUMAN**	Beta-2-glycoprotein 1 OS=Homo sapiens GN=APOH PE=1 SV=3	38298	ND	40.68	0	1	0.00	0.41	0.20
**P04220|MUCB_HUMAN**	Ig mu heavy chain disease protein OS=Homo sapiens PE=1 SV=1	43057	66.98	ND	1	0	0.34	0.00	0.17
**O14791|APOL1_HUMAN**	Apolipoprotein L1 OS=Homo sapiens GN=APOL1 PE=1 SV=5	43974	70.78	ND	1	0	0.34	0.00	0.17
**P06727|APOA4_HUMAN**	Apolipoprotein A-IV OS=Homo sapiens GN=APOA4 PE=1 SV=3	45399	93.53	ND	1	0	0.33	0.00	0.16
**P10643|CO7_HUMAN**	Complement component C7 OS=Homo sapiens GN=C7 PE=1 SV=2	93518	134.15	ND	2	0	0.32	0.00	0.16
**P01871|IGHM_HUMAN**	Ig mu chain C region OS=Homo sapiens GN=IGHM PE=1 SV=3	49307	66.98	ND	1	0	0.30	0.00	0.15
**P19827|ITIH1_HUMAN**	Inter-alpha-trypsin inhibitor heavy chain H1 OS=Homo sapiens GN=ITIH1 PE=1 SV=3	101389	134.3	61.3	1	1	0.15	0.15	0.15
**P07225|PROS_HUMAN**	Vitamin K-dependent protein S OS=Homo sapiens GN=PROS1 PE=1 SV=1	75123	26.24	ND	1	0	0.20	0.00	0.10
**P09871|C1S_HUMAN**	Complement C1s subcomponent OS=Homo sapiens GN=C1S PE=1 SV=1	76685	118.12	ND	1	0	0.19	0.00	0.10
**P12259|FA5_HUMAN**	Coagulation factor V OS=Homo sapiens GN=F5 PE=1 SV=4	251701	35.03	ND	1	0	0.06	0.00	0.03
**O00763|ACACB_HUMAN**	Acetyl-CoA carboxylase 2 OS=Homo sapiens GN=ACACB PE=1 SV=3	276539	27.59	ND	1	0	0.05	0.00	0.03
**O14686|MLL2_HUMAN**	Histone-lysine N-methyltransferase MLL2 OS=Homo sapiens GN=MLL2 PE=1 SV=2	593399	40.18	ND	1	0	0.02	0.00	0.01

Table S1b: (8% EMT + 10% human plasma)

Accession	Description	Mw	-10lgP S 1	-10lgP S 2	Spectra S 1	Spectra S 2	NpSpCk S 1	NpSpCk S 2	Average NpSpCk
**P01857|IGHG1_HUMAN**	Ig gamma-1 chain C region OS=Homo sapiens GN=IGHG1 PE=1 SV=1	36106	305.32	333.86	28	33	11.45	8.26	9.85
**P01861|IGHG4_HUMAN**	Ig gamma-4 chain C region OS=Homo sapiens GN=IGHG4 PE=1 SV=1	35941	314.59	319.56	25	23	10.27	5.78	8.02
**P02675|FIBB_HUMAN**	Fibrinogen beta chain OS=Homo sapiens GN=FGB PE=1 SV=2	55928	286.85	424.54	24	49	6.33	7.91	7.12
**P02679|FIBG_HUMAN**	Fibrinogen gamma chain OS=Homo sapiens GN=FGG PE=1 SV=3	51512	305.08	342.69	20	32	5.73	5.61	5.67
**P02768|ALBU_HUMAN**	Serum albumin OS=Homo sapiens GN=ALB PE=1 SV=2	69367	320.81	357.2	25	41	5.32	5.34	5.33
**P01859|IGHG2_HUMAN**	Ig gamma-2 chain C region OS=Homo sapiens GN=IGHG2 PE=1 SV=2	35901	247.19	261.31	14	12	5.76	3.02	4.39
**P04196|HRG_HUMAN**	Histidine-rich glycoprotein OS=Homo sapiens GN=HRG PE=1 SV=1	59578	270.99	282.68	17	21	4.21	3.18	3.70
**P02671|FIBA_HUMAN**	Fibrinogen alpha chain OS=Homo sapiens GN=FGA PE=1 SV=2	94973	302	375.18	19	46	2.95	4.38	3.66
**P02656|APOC3_HUMAN**	Apolipoprotein C-III OS=Homo sapiens GN=APOC3 PE=1 SV=1	10852	138.04	105.01	3	3	4.08	2.50	3.29
**P01024|CO3_HUMAN**	Complement C3 OS=Homo sapiens GN=C3 PE=1 SV=2	187147	427.91	489.53	36	64	2.84	3.09	2.96
**P01860|IGHG3_HUMAN**	Ig gamma-3 chain C region OS=Homo sapiens GN=IGHG3 PE=1 SV=2	41287	ND	286.63	0	22	0.00	4.81	2.41
**P02647|APOA1_HUMAN**	Apolipoprotein A-I OS=Homo sapiens GN=APOA1 PE=1 SV=1	30778	223.08	223.34	6	7	2.88	2.05	2.47
**P0CG06|LAC3_HUMAN**	Ig lambda-3 chain C regions OS=Homo sapiens GN=IGLC3 PE=1 SV=1	11237	94.25	180.83	1	3	1.31	2.41	1.86
**P0CG05|LAC2_HUMAN**	Ig lambda-2 chain C regions OS=Homo sapiens GN=IGLC2 PE=1 SV=1	11294	94.25	180.83	1	3	1.31	2.40	1.85
**P36955|PEDF_HUMAN**	Pigment epithelium-derived factor OS=Homo sapiens GN=SERPINF1 PE=1 SV=4	46312	161.59	183.12	6	7	1.91	1.37	1.64
**P04114|APOB_HUMAN**	Apolipoprotein B-100 OS=Homo sapiens GN=APOB PE=1 SV=2	515611	478.69	458.39	76	61	2.18	1.07	1.62
**P0C0L4|CO4A_HUMAN**	Complement C4-A OS=Homo sapiens GN=C4A PE=1 SV=1	192770	287.29	326.77	17	31	1.30	1.45	1.38
**P0C0L5|CO4B_HUMAN**	Complement C4-B OS=Homo sapiens GN=C4B PE=1 SV=1	192792	287.29	326.77	17	31	1.30	1.45	1.38
**P02655|APOC2_HUMAN**	Apolipoprotein C-II OS=Homo sapiens GN=APOC2 PE=1 SV=1	11284	126.19	ND	2	0	2.62	0.00	1.31
**P01834|IGKC_HUMAN**	Ig kappa chain C region OS=Homo sapiens GN=IGKC PE=1 SV=1	11609	146.13	ND	2	0	2.54	0.00	1.27
**P06727|APOA4_HUMAN**	Apolipoprotein A-IV OS=Homo sapiens GN=APOA4 PE=1 SV=3	45399	93.53	252.5	1	10	0.33	1.99	1.16
**P02766|TTHY_HUMAN**	Transthyretin OS=Homo sapiens GN=TTR PE=1 SV=1	15887	91.37	91.83	1	2	0.93	1.14	1.03
**P04004|VTNC_HUMAN**	Vitronectin OS=Homo sapiens GN=VTN PE=1 SV=1	54306	145.23	213.09	2	8	0.54	1.33	0.94
**P00751|CFAB_HUMAN**	Complement factor B OS=Homo sapiens GN=CFB PE=1 SV=2	85533	199.46	203.27	6	8	1.04	0.84	0.94

**P01625|KV402_HUMAN**	Ig kappa chain V-IV region Len OS=Homo sapiens PE=1 SV=2	12640	110.79	93.9	1	1	1.17	0.71	0.94
**P06312|KV401_HUMAN**	Ig kappa chain V-IV region (Fragment) OS=Homo sapiens GN=IGKV4-1 PE=4 SV=1	13380	110.79	93.9	1	1	1.10	0.68	0.89
**P01031|CO5_HUMAN**	Complement C5 OS=Homo sapiens GN=C5 PE=1 SV=4	188304	252.48	206.24	15	12	1.18	0.58	0.88
**P07360|CO8G_HUMAN**	Complement component C8 gamma chain OS=Homo sapiens GN=C8G PE=1 SV=3	22277	ND	148.54	0	4	0.00	1.62	0.81
**P06313|KV403_HUMAN**	Ig kappa chain V-IV region JI OS=Homo sapiens PE=4 SV=1	14633	110.79	93.9	1	1	1.01	0.62	0.81
**P06314|KV404_HUMAN**	Ig kappa chain V-IV region B17 OS=Homo sapiens PE=2 SV=1	14966	110.79	93.9	1	1	0.99	0.60	0.79
**P04003|C4BPA_HUMAN**	C4b-binding protein alpha chain OS=Homo sapiens GN=C4BPA PE=1 SV=2	67033	117.73	201.72	2	8	0.44	1.08	0.76
**P00747|PLMN_HUMAN**	Plasminogen OS=Homo sapiens GN=PLG PE=1 SV=2	90569	165.56	190.25	5	7	0.81	0.70	0.76
**P01876|IGHA1_HUMAN**	Ig alpha-1 chain C region OS=Homo sapiens GN=IGHA1 PE=1 SV=2	37655	36.1	121.35	2	3	0.78	0.72	0.75
**P01871|IGHM_HUMAN**	Ig mu chain C region OS=Homo sapiens GN=IGHM PE=1 SV=3	49307	66.98	148.69	1	6	0.30	1.10	0.70
**P02652|APOA2_HUMAN**	Apolipoprotein A-II OS=Homo sapiens GN=APOA2 PE=1 SV=1	11175	55.8	ND	1	0	1.32	0.00	0.66
**P0CF74|LAC6_HUMAN**	Ig lambda-6 chain C region OS=Homo sapiens GN=IGLC6 PE=4 SV=1	11277	94.25	ND	1	0	1.31	0.00	0.65
**A0M8Q6|LAC7_HUMAN**	Ig lambda-7 chain C region OS=Homo sapiens GN=IGLC7 PE=1 SV=2	11303	94.25	ND	1	0	1.31	0.00	0.65
**P02649|APOE_HUMAN**	Apolipoprotein E OS=Homo sapiens GN=APOE PE=1 SV=1	36154	ND	213.8	0	5	0.00	1.25	0.62
**P0CG04|LAC1_HUMAN**	Ig lambda-1 chain C regions OS=Homo sapiens GN=IGLC1 PE=1 SV=1	11348	94.25	ND	1	0	1.30	0.00	0.65
**P02749|APOH_HUMAN**	Beta-2-glycoprotein 1 OS=Homo sapiens GN=APOH PE=1 SV=3	38298	ND	110.71	0	5	0.00	1.18	0.59
**P02745|C1QA_HUMAN**	Complement C1q subcomponent subunit A OS=Homo sapiens GN=C1QA PE=1 SV=2	26017	127	ND	2	0	1.13	0.00	0.57
**P08603|CFAH_HUMAN**	Complement factor H OS=Homo sapiens GN=CFH PE=1 SV=4	139096	166.03	186.4	4	10	0.42	0.65	0.54
**P02751|FINC_HUMAN**	Fibronectin OS=Homo sapiens GN=FN1 PE=1 SV=4	262622	242.97	340.65	8	17	0.45	0.58	0.52
**P19823|ITIH2_HUMAN**	Inter-alpha-trypsin inhibitor heavy chain H2 OS=Homo sapiens GN=ITIH2 PE=1 SV=2	106463	140.44	180.03	4	5	0.55	0.42	0.49
**P01042|KNG1_HUMAN**	Kininogen-1 OS=Homo sapiens GN=KNG1 PE=1 SV=2	71957	ND	207.86	0	7	0.00	0.88	0.44
**P01621|KV303_HUMAN**	Ig kappa chain V-III region NG9 (Fragment) OS=Homo sapiens PE=1 SV=1	10729	ND	109.67	0	1	0.00	0.84	0.42
**P07358|CO8B_HUMAN**	Complement component C8 beta chain OS=Homo sapiens GN=C8B PE=1 SV=3	67047	78.34	125.64	2	3	0.44	0.40	0.42
**P01877|IGHA2_HUMAN**	Ig alpha-2 chain C region OS=Homo sapiens GN=IGHA2 PE=1 SV=3	36526	36.1	ND	2	0	0.81	0.00	0.40
**P09871|C1S_HUMAN**	Complement C1s subcomponent OS=Homo sapiens GN=C1S PE=1 SV=1	76685	118.12	143.03	1	5	0.19	0.59	0.39
**P80362|KV125_HUMAN**	Ig kappa chain V-I region WAT OS=Homo sapiens PE=1 SV=1	11737	ND	55.09	0	1	0.00	0.77	0.38
**P01623|KV305_HUMAN**	Ig kappa chain V-III region WOL OS=Homo sapiens PE=1 SV=1	11746	ND	109.67	0	1	0.00	0.77	0.38
**P01620|KV302_HUMAN**	Ig kappa chain V-III region SIE OS=Homo sapiens PE=1 SV=1	11775	ND	109.67	0	1	0.00	0.77	0.38

**P01622|KV304_HUMAN**	Ig kappa chain V-III region Ti OS=Homo sapiens PE=1 SV=1	11788	ND	109.67	0	1	0.00	0.77	0.38
**P04206|KV307_HUMAN**	Ig kappa chain V-III region GOL OS=Homo sapiens PE=1 SV=1	11830	ND	109.67	0	1	0.00	0.76	0.38
**P04433|KV309_HUMAN**	Ig kappa chain V-III region VG (Fragment) OS=Homo sapiens PE=1 SV=1	12575	ND	88.55	0	1	0.00	0.72	0.36
**P01777|HV316_HUMAN**	Ig heavy chain V-III region TEI OS=Homo sapiens PE=1 SV=1	12802	ND	103.45	0	1	0.00	0.71	0.35
**P02790|HEMO_HUMAN**	Hemopexin OS=Homo sapiens GN=HPX PE=1 SV=2	51676	ND	107.2	0	4	0.00	0.70	0.35
**P01766|HV305_HUMAN**	Ig heavy chain V-III region BRO OS=Homo sapiens PE=1 SV=1	13227	ND	103.45	0	1	0.00	0.68	0.34
**P18135|KV312_HUMAN**	Ig kappa chain V-III region HAH OS=Homo sapiens PE=2 SV=1	14073	ND	109.67	0	1	0.00	0.64	0.32
**P18136|KV313_HUMAN**	Ig kappa chain V-III region HIC OS=Homo sapiens PE=2 SV=2	14089	ND	109.67	0	1	0.00	0.64	0.32
**P04207|KV308_HUMAN**	Ig kappa chain V-III region CLL OS=Homo sapiens PE=1 SV=2	14275	ND	94.16	0	1	0.00	0.63	0.32
**P10909|CLUS_HUMAN**	Clusterin OS=Homo sapiens GN=CLU PE=1 SV=1	52495	69.78	85.41	1	2	0.28	0.34	0.31
**B9A064|IGLL5_HUMAN**	Immunoglobulin lambda-like polypeptide 5 OS=Homo sapiens GN=IGLL5 PE=2 SV=2	23063	94.25	ND	1	0	0.64	0.00	0.32
**Q14624|ITIH4_HUMAN**	Inter-alpha-trypsin inhibitor heavy chain H4 OS=Homo sapiens GN=ITIH4 PE=1 SV=4	103357	ND	199.99	0	7	0.00	0.61	0.31
**P02760|AMBP_HUMAN**	Protein AMBP OS=Homo sapiens GN=AMBP PE=1 SV=1	39000	69.18	78.19	1	1	0.38	0.23	0.31
**P10643|CO7_HUMAN**	Complement component C7 OS=Homo sapiens GN=C7 PE=1 SV=2	93518	134.15	130.92	2	3	0.32	0.29	0.30
**P00736|C1R_HUMAN**	Complement C1r subcomponent OS=Homo sapiens GN=C1R PE=1 SV=2	80119	88.75	82.22	2	2	0.37	0.23	0.30
**P02748|CO9_HUMAN**	Complement component C9 OS=Homo sapiens GN=C9 PE=1 SV=2	63173	ND	145.99	0	4	0.00	0.57	0.29
**P13671|CO6_HUMAN**	Complement component C6 OS=Homo sapiens GN=C6 PE=1 SV=3	104786	107.14	120.99	2	3	0.28	0.26	0.27
**P06681|CO2_HUMAN**	Complement C2 OS=Homo sapiens GN=C2 PE=1 SV=2	83268	ND	89.58	0	4	0.00	0.43	0.22
**P07225|PROS_HUMAN**	Vitamin K-dependent protein S OS=Homo sapiens GN=PROS1 PE=1 SV=1	75123	26.24	66.88	1	2	0.20	0.24	0.22
**P00748|FA12_HUMAN**	Coagulation factor XII OS=Homo sapiens GN=F12 PE=1 SV=3	67792	ND	114.74	0	3	0.00	0.40	0.20
**P04220|MUCB_HUMAN**	Ig mu heavy chain disease protein OS=Homo sapiens PE=1 SV=1	43057	66.98	ND	1	0	0.34	0.00	0.17
**O14791|APOL1_HUMAN**	Apolipoprotein L1 OS=Homo sapiens GN=APOL1 PE=1 SV=5	43974	70.78	ND	1	0	0.34	0.00	0.17
**P35030|TRY3_HUMAN**	Trypsin-3 OS=Homo sapiens GN=PRSS3 PE=1 SV=2	32529	ND	34.41	0	1	0.00	0.28	0.14
**P00739|HPTR_HUMAN**	Haptoglobin-related protein OS=Homo sapiens GN=HPR PE=1 SV=2	39030	ND	50.6	0	1	0.00	0.23	0.12
**Q96PD5|PGRP2_HUMAN**	N-acetylmuramoyl-L-alanine amidase OS=Homo sapiens GN=PGLYRP2 PE=1 SV=1	62217	59.02	ND	1	0	0.24	0.00	0.12
**P03951|FA11_HUMAN**	Coagulation factor XI OS=Homo sapiens GN=F11 PE=1 SV=1	70109	69.62	ND	1	0	0.21	0.00	0.11
**P15169|CBPN_HUMAN**	Carboxypeptidase N catalytic chain OS=Homo sapiens GN=CPN1 PE=1 SV=1	52286	ND	68.83	0	1	0.00	0.17	0.09
**P19827|ITIH1_HUMAN**	Inter-alpha-trypsin inhibitor heavy chain H1 OS=Homo sapiens GN=ITIH1 PE=1 SV=3	101389	134.3	ND	1	0	0.15	0.00	0.07

**P07357|CO8A_HUMAN**	Complement component C8 alpha chain OS=Homo sapiens GN=C8A PE=1 SV=2	65163	ND	78.69	0	1	0.00	0.14	0.07
**P03952|KLKB1_HUMAN**	Plasma kallikrein OS=Homo sapiens GN=KLKB1 PE=1 SV=1	71370	ND	54.25	0	1	0.00	0.13	0.06
**P02787|TRFE_HUMAN**	Serotransferrin OS=Homo sapiens GN=TF PE=1 SV=3	77064	ND	101.1	0	1	0.00	0.12	0.06
**P06396|GELS_HUMAN**	Gelsolin OS=Homo sapiens GN=GSN PE=1 SV=1	85697	ND	48.75	0	1	0.00	0.11	0.05
**P00450|CERU_HUMAN**	Ceruloplasmin OS=Homo sapiens GN=CP PE=1 SV=1	122205	ND	81.05	0	1	0.00	0.07	0.04
**P12259|FA5_HUMAN**	Coagulation factor V OS=Homo sapiens GN=F5 PE=1 SV=4	251701	35.03	ND	1	0	0.06	0.00	0.03
**O00763|ACACB_HUMAN**	Acetyl-CoA carboxylase 2 OS=Homo sapiens GN=ACACB PE=1 SV=3	276539	27.59	ND	1	0	0.05	0.00	0.03
**O14686|MLL2_HUMAN**	Histone-lysine N-methyltransferase MLL2 OS=Homo sapiens GN=MLL2 PE=1 SV=2	593399	40.18	ND	1	0	0.02	0.00	0.01

Table S1c: (12% EMT + 10% human plasma)

Accession	Description	Mw	-10lgP S 1	-10lgP S 2	Spectra S 1	Spectra S 2	NpSpCk S 1	NpSpCk S 2	Average NpSpCk
**P01857|IGHG1_HUMAN**	Ig gamma-1 chain C region OS=Homo sapiens GN=IGHG1 PE=1 SV=1	36106	285.92	276.43	27	22	10.77	9.38	10.08
**P01861|IGHG4_HUMAN**	Ig gamma-4 chain C region OS=Homo sapiens GN=IGHG4 PE=1 SV=1	35941	275.34	264.3	24	19	9.62	8.14	8.88
**P02768|ALBU_HUMAN**	Serum albumin OS=Homo sapiens GN=ALB PE=1 SV=2	69367	326.01	356.07	28	38	5.81	8.43	7.12
**P01860|IGHG3_HUMAN**	Ig gamma-3 chain C region OS=Homo sapiens GN=IGHG3 PE=1 SV=2	41287	249.46	229.66	22	16	7.68	5.97	6.82
**P02675|FIBB_HUMAN**	Fibrinogen beta chain OS=Homo sapiens GN=FGB PE=1 SV=2	55928	298.78	315.55	26	25	6.70	6.88	6.79
**P04196|HRG_HUMAN**	Histidine-rich glycoprotein OS=Homo sapiens GN=HRG PE=1 SV=1	59578	278.98	267.99	20	23	4.84	5.94	5.39
**P02679|FIBG_HUMAN**	Fibrinogen gamma chain OS=Homo sapiens GN=FGG PE=1 SV=3	51512	289.34	301.74	18	18	5.03	5.38	5.21
**P01024|CO3_HUMAN**	Complement C3 OS=Homo sapiens GN=C3 PE=1 SV=2	187147	456.4	464.92	57	56	4.39	4.61	4.50
**P02656|APOC3_HUMAN**	Apolipoprotein C-III OS=Homo sapiens GN=APOC3 PE=1 SV=1	10852	135.79	150.56	2	4	2.65	5.67	4.16
**P01859|IGHG2_HUMAN**	Ig gamma-2 chain C region OS=Homo sapiens GN=IGHG2 PE=1 SV=2	35901	231.2	200.09	11	8	4.41	3.43	3.92
**P02671|FIBA_HUMAN**	Fibrinogen alpha chain OS=Homo sapiens GN=FGA PE=1 SV=2	94973	311.45	271.78	26	17	3.94	2.76	3.35
**P02766|TTHY_HUMAN**	Transthyretin OS=Homo sapiens GN=TTR PE=1 SV=1	15887	120.94	161.09	2	4	1.81	3.88	2.84
**P02647|APOA1_HUMAN**	Apolipoprotein A-I OS=Homo sapiens GN=APOA1 PE=1 SV=1	30778	230.72	210.81	5	6	2.34	3.00	2.67
**P0C0L4|CO4A_HUMAN**	Complement C4-A OS=Homo sapiens GN=C4A PE=1 SV=1	192770	318.53	322.58	19	21	1.42	1.68	1.55
**P0C0L5|CO4B_HUMAN**	Complement C4-B OS=Homo sapiens GN=C4B PE=1 SV=1	192792	318.53	322.58	19	21	1.42	1.68	1.55
**P06727|APOA4_HUMAN**	Apolipoprotein A-IV OS=Homo sapiens GN=APOA4 PE=1 SV=3	45399	156.37	152.55	5	4	1.59	1.36	1.47
**P0CG06|LAC3_HUMAN**	Ig lambda-3 chain C regions OS=Homo sapiens GN=IGLC3 PE=1 SV=1	11237	93.04	80.46	1	1	1.28	1.37	1.33
**P0CF74|LAC6_HUMAN**	Ig lambda-6 chain C region OS=Homo sapiens GN=IGLC6 PE=4 SV=1	11277	93.04	80.46	1	1	1.28	1.36	1.32
**P0CG05|LAC2_HUMAN**	Ig lambda-2 chain C regions OS=Homo sapiens GN=IGLC2 PE=1 SV=1	11294	93.04	80.46	1	1	1.28	1.36	1.32
**A0M8Q6|LAC7_HUMAN**	Ig lambda-7 chain C region OS=Homo sapiens GN=IGLC7 PE=1 SV=2	11303	93.04	80.46	1	1	1.27	1.36	1.32
**P0CG04|LAC1_HUMAN**	Ig lambda-1 chain C regions OS=Homo sapiens GN=IGLC1 PE=1 SV=1	11348	93.04	80.46	1	1	1.27	1.36	1.31
**P36955|PEDF_HUMAN**	Pigment epithelium-derived factor OS=Homo sapiens GN=SERPINF1 PE=1 SV=4	46312	190.88	83.65	5	2	1.56	0.66	1.11
**P00747|PLMN_HUMAN**	Plasminogen OS=Homo sapiens GN=PLG PE=1 SV=2	90569	145.51	177.17	6	7	0.95	1.19	1.07
**P00751|CFAB_HUMAN**	Complement factor B OS=Homo sapiens GN=CFB PE=1 SV=2	85533	183.64	207.3	6	6	1.01	1.08	1.05
**P01031|CO5_HUMAN**	Complement C5 OS=Homo sapiens GN=C5 PE=1 SV=4	188304	260.64	214.51	14	12	1.07	0.98	1.03
**P04114|APOB_HUMAN**	Apolipoprotein B-100 OS=Homo sapiens GN=APOB PE=1 SV=2	515611	394.74	381.97	37	32	1.03	0.96	0.99

**P04003|C4BPA_HUMAN**	C4b-binding protein alpha chain OS=Homo sapiens GN=C4BPA PE=1 SV=2	67033	122.85	77.62	5	3	1.07	0.69	0.88
**P19823|ITIH2_HUMAN**	Inter-alpha-trypsin inhibitor heavy chain H2 OS=Homo sapiens GN=ITIH2 PE=1 SV=2	106463	197.27	148.17	6	5	0.81	0.72	0.77
**P02652|APOA2_HUMAN**	Apolipoprotein A-II OS=Homo sapiens GN=APOA2 PE=1 SV=1	11175	58.52	ND	1	0	1.29	0.00	0.64
**B9A064|IGLL5_HUMAN**	Immunoglobulin lambda-like polypeptide 5 OS=Homo sapiens GN=IGLL5 PE=2 SV=2	23063	93.04	80.46	1	1	0.62	0.67	0.65
**P01834|IGKC_HUMAN**	Ig kappa chain C region OS=Homo sapiens GN=IGKC PE=1 SV=1	11609	85.98	ND	1	0	1.24	0.00	0.62
**P10909|CLUS_HUMAN**	Clusterin OS=Homo sapiens GN=CLU PE=1 SV=1	52495	82.6	144.9	1	3	0.27	0.88	0.58
**P04004|VTNC_HUMAN**	Vitronectin OS=Homo sapiens GN=VTN PE=1 SV=1	54306	141.22	143.36	2	2	0.53	0.57	0.55
**P01042|KNG1_HUMAN**	Kininogen-1 OS=Homo sapiens GN=KNG1 PE=1 SV=2	71957	ND	155.38	0	5	0.00	1.07	0.53
**P07358|CO8B_HUMAN**	Complement component C8 beta chain OS=Homo sapiens GN=C8B PE=1 SV=3	67047	86.59	106.41	2	2	0.43	0.46	0.44
**P05546|HEP2_HUMAN**	Heparin cofactor 2 OS=Homo sapiens GN=SERPIND1 PE=1 SV=3	57071	ND	86.67	0	3	0.00	0.81	0.40
**P02751|FINC_HUMAN**	Fibronectin OS=Homo sapiens GN=FN1 PE=1 SV=4	262622	205.71	200.08	8	5	0.44	0.29	0.37
**P06681|CO2_HUMAN**	Complement C2 OS=Homo sapiens GN=C2 PE=1 SV=2	83268	55.19	116.93	2	2	0.35	0.37	0.36
**P35858|ALS_HUMAN**	Insulin-like growth factor-binding protein complex acid labile subunit OS=Homo sapiens GN=IGFALS PE=1 SV=1	66035	116.94	80.08	2	1	0.44	0.23	0.33
**P08603|CFAH_HUMAN**	Complement factor H OS=Homo sapiens GN=CFH PE=1 SV=4	139096	105.01	91.97	4	2	0.41	0.22	0.32
**P02790|HEMO_HUMAN**	Hemopexin OS=Homo sapiens GN=HPX PE=1 SV=2	51676	ND	63.24	0	2	0.00	0.60	0.30
**P02745|C1QA_HUMAN**	Complement C1q subcomponent subunit A OS=Homo sapiens GN=C1QA PE=1 SV=2	26017	ND	61	0	1	0.00	0.59	0.30
**P02748|CO9_HUMAN**	Complement component C9 OS=Homo sapiens GN=C9 PE=1 SV=2	63173	64.94	ND	2	0	0.46	0.00	0.23
**P07357|CO8A_HUMAN**	Complement component C8 alpha chain OS=Homo sapiens GN=C8A PE=1 SV=2	65163	57.56	65.33	1	1	0.22	0.24	0.23
**P00748|FA12_HUMAN**	Coagulation factor XII OS=Homo sapiens GN=F12 PE=1 SV=3	67792	ND	54.17	0	2	0.00	0.45	0.23
**P02649|APOE_HUMAN**	Apolipoprotein E OS=Homo sapiens GN=APOE PE=1 SV=1	36154	ND	95.48	0	1	0.00	0.43	0.21
**P13671|CO6_HUMAN**	Complement component C6 OS=Homo sapiens GN=C6 PE=1 SV=3	104786	138.32	ND	3	0	0.41	0.00	0.21
**P03952|KLKB1_HUMAN**	Plasma kallikrein OS=Homo sapiens GN=KLKB1 PE=1 SV=1	71370	111.69	ND	2	0	0.40	0.00	0.20
**P01877|IGHA2_HUMAN**	Ig alpha-2 chain C region OS=Homo sapiens GN=IGHA2 PE=1 SV=3	36526	25.83	ND	1	0	0.39	0.00	0.20
**P09871|C1S_HUMAN**	Complement C1s subcomponent OS=Homo sapiens GN=C1S PE=1 SV=1	76685	67.29	70.86	1	1	0.19	0.20	0.19
**P01876|IGHA1_HUMAN**	Ig alpha-1 chain C region OS=Homo sapiens GN=IGHA1 PE=1 SV=2	37655	25.83	ND	1	0	0.38	0.00	0.19
**P02749|APOH_HUMAN**	Beta-2-glycoprotein 1 OS=Homo sapiens GN=APOH PE=1 SV=3	38298	32.38	ND	1	0	0.38	0.00	0.19
**Q9Y2T7|YBOX2_HUMAN**	Y-box-binding protein 2 OS=Homo sapiens GN=YBX2 PE=1 SV=2	38518	22.36	ND	1	0	0.37	0.00	0.19
**P04220|MUCB_HUMAN**	Ig mu heavy chain disease protein OS=Homo sapiens PE=1 SV=1	43057	67.72	ND	1	0	0.33	0.00	0.17
**P10643|CO7_HUMAN**	Complement component C7 OS=Homo sapiens GN=C7 PE=1 SV=2	93518	137.25	ND	2	0	0.31	0.00	0.15

**P01871|IGHM_HUMAN**	Ig mu chain C region OS=Homo sapiens GN=IGHM PE=1 SV=3	49307	67.72	ND	1	0	0.29	0.00	0.15
**P19827|ITIH1_HUMAN**	Inter-alpha-trypsin inhibitor heavy chain H1 OS=Homo sapiens GN=ITIH1 PE=1 SV=3	101389	144.86	138.38	1	1	0.14	0.15	0.15
**P02774|VTDB_HUMAN**	Vitamin D-binding protein OS=Homo sapiens GN=GC PE=1 SV=1	52964	74	ND	1	0	0.27	0.00	0.14
**P18428|LBP_HUMAN**	Lipopolysaccharide-binding protein OS=Homo sapiens GN=LBP PE=1 SV=3	53384	45.85	ND	1	0	0.27	0.00	0.13
**Q96PD5|PGRP2_HUMAN**	N-acetylmuramoyl-L-alanine amidase OS=Homo sapiens GN=PGLYRP2 PE=1 SV=1	62217	ND	49.32	0	1	0.00	0.25	0.12
**P05156|CFAI_HUMAN**	Complement factor I OS=Homo sapiens GN=CFI PE=1 SV=2	65750	ND	90.8	0	1	0.00	0.23	0.12
**P03951|FA11_HUMAN**	Coagulation factor XI OS=Homo sapiens GN=F11 PE=1 SV=1	70109	64.9	ND	1	0	0.21	0.00	0.10
**A1IGU5|ARH37_HUMAN**	Rho guanine nucleotide exchange factor 37 OS=Homo sapiens GN=ARHGEF37 PE=2 SV=2	76278	20.48	ND	1	0	0.19	0.00	0.09
**P02787|TRFE_HUMAN**	Serotransferrin OS=Homo sapiens GN=TF PE=1 SV=3	77064	100.03	ND	1	0	0.19	0.00	0.09
**Q9UPX6|K1024_HUMAN**	UPF0258 protein KIAA1024 OS=Homo sapiens GN=KIAA1024 PE=2 SV=3	102993	21.97	ND	1	0	0.14	0.00	0.07
**P12259|FA5_HUMAN**	Coagulation factor V OS=Homo sapiens GN=F5 PE=1 SV=4	251701	39.27	ND	2	0	0.11	0.00	0.06
**O95714|HERC2_HUMAN**	E3 ubiquitin-protein ligase HERC2 OS=Homo sapiens GN=HERC2 PE=1 SV=2	527234	ND	27.95	0	1	0.00	0.03	0.01

Table S1d: (4% EMT + 100% human plasma)

Accession	Description	Mw	-10lgP		Spectra		NpSpCk		Average NpSpCk
			S 1	S 2	S 1	S 2	S 1	S 2	
**P02656|APOC3_HUMAN**	Apolipoprotein C-III OS=Homo sapiens GN=APOC3 PE=1 SV=1	10852	170.23	194.5	6	6	32.00	29.12	30.56
**P02768|ALBU_HUMAN**	Serum albumin OS=Homo sapiens GN=ALB PE=1 SV=2	69367	339.13	375.48	15	18	12.52	13.67	13.09
**P02671|FIBA_HUMAN**	Fibrinogen alpha chain OS=Homo sapiens GN=FGA PE=1 SV=2	94973	287.01	329.76	19	22	11.58	12.20	11.89
**P02679|FIBG_HUMAN**	Fibrinogen gamma chain OS=Homo sapiens GN=FGG PE=1 SV=3	51512	269.57	302.63	9	12	10.11	12.27	11.19
**P02675|FIBB_HUMAN**	Fibrinogen beta chain OS=Homo sapiens GN=FGB PE=1 SV=2	55928	234.04	244.47	7	9	7.24	8.48	7.86
**P02647|APOA1_HUMAN**	Apolipoprotein A-I OS=Homo sapiens GN=APOA1 PE=1 SV=1	30778	151.25	160.63	4	2	7.52	3.42	5.47
**P02766|TTHY_HUMAN**	Transthyretin OS=Homo sapiens GN=TTR PE=1 SV=1	15887	42.56	42.11	1	1	3.64	3.32	3.48
**P19823|ITIH2_HUMAN**	Inter-alpha-trypsin inhibitor heavy chain H2 OS=Homo sapiens GN=ITIH2 PE=1 SV=2	106463	201.35	155.98	6	4	3.26	1.98	2.62
**P01024|CO3_HUMAN**	Complement C3 OS=Homo sapiens GN=C3 PE=1 SV=2	187147	289.51	369.3	6	12	1.86	3.38	2.62
**P04004|VTNC_HUMAN**	Vitronectin OS=Homo sapiens GN=VTN PE=1 SV=1	54306	145.59	136.06	2	2	2.13	1.94	2.04
**P09871|C1S_HUMAN**	Complement C1s subcomponent OS=Homo sapiens GN=C1S PE=1 SV=1	76685	ND	202.64	0	5	0.00	3.43	1.72
**P04114|APOB_HUMAN**	Apolipoprotein B-100 OS=Homo sapiens GN=APOB PE=1 SV=2	515611	336	394.38	14	16	1.57	1.63	1.60
**P01042|KNG1_HUMAN**	Kininogen-1 OS=Homo sapiens GN=KNG1 PE=1 SV=2	71957	74.91	66.89	2	2	1.61	1.46	1.54
**P03952|KLKB1_HUMAN**	Plasma kallikrein OS=Homo sapiens GN=KLKB1 PE=1 SV=1	71370	73.02	ND	2	0	1.62	0.00	0.81
**P02787|TRFE_HUMAN**	Serotransferrin OS=Homo sapiens GN=TF PE=1 SV=3	77064	136.08	124.28	1	1	0.75	0.68	0.72
**P00736|C1R_HUMAN**	Complement C1r subcomponent OS=Homo sapiens GN=C1R PE=1 SV=2	80119	117.32	102.01	1	1	0.72	0.66	0.69
**P02749|APOH_HUMAN**	Beta-2-glycoprotein 1 OS=Homo sapiens GN=APOH PE=1 SV=3	38298	ND	44.25	0	1	0.00	1.38	0.69
**P04196|HRG_HUMAN**	Histidine-rich glycoprotein OS=Homo sapiens GN=HRG PE=1 SV=1	59578	42.65	ND	1	0	0.97	0.00	0.49
**P00450|CERU_HUMAN**	Ceruloplasmin OS=Homo sapiens GN=CP PE=1 SV=1	122205	123.11	ND	1	0	0.47	0.00	0.24
**P08603|CFAH_HUMAN**	Complement factor H OS=Homo sapiens GN=CFH PE=1 SV=4	139096	43.38	ND	1	0	0.42	0.00	0.21
**P0C0L4|CO4A_HUMAN**	Complement C4-A OS=Homo sapiens GN=C4A PE=1 SV=1	192770	ND	54.3	0	1	0.00	0.27	0.14
**P0C0L5|CO4B_HUMAN**	Complement C4-B OS=Homo sapiens GN=C4B PE=1 SV=1	192792	ND	54.3	0	1	0.00	0.27	0.14
**Q5VT25|MRCKA_HUMAN**	Serine/threonine-protein kinase MRCK alpha OS=Homo sapiens GN=CDC42BPA PE=1 SV=1	197306	ND	38.23	0	1	0.00	0.27	0.13
**Q8IYW2|TTC40_HUMAN**	Tetratricopeptide repeat protein 40 OS=Homo sapiens GN=TTC40 PE=2 SV=3	303499	ND	38.94	0	1	0.00	0.17	0.09

Table S1e: (8% EMT + 100% human plasma)

Accession	Description	Mw	-10lgP S 1	-10lgP S 2	Spectra S 1	Spectra S 2	NpSpCk S 1	NpSpCk S 2	Average NpSpCk
**P02679|FIBG_HUMAN**	Fibrinogen gamma chain OS=Homo sapiens GN=FGG PE=1 SV=3	51512	303.34	307.17	13	19	13.29	21.35	17.32
**P02671|FIBA_HUMAN**	Fibrinogen alpha chain OS=Homo sapiens GN=FGA PE=1 SV=2	94973	313.54	272.41	25	27	13.86	16.46	15.16
**P02768|ALBU_HUMAN**	Serum albumin OS=Homo sapiens GN=ALB PE=1 SV=2	69367	318.29	308.02	13	19	9.87	15.85	12.86
**P02675|FIBB_HUMAN**	Fibrinogen beta chain OS=Homo sapiens GN=FGB PE=1 SV=2	55928	228.16	225.19	9	16	8.47	16.56	12.52
**P02656|APOC3_HUMAN**	Apolipoprotein C-III OS=Homo sapiens GN=APOC3 PE=1 SV=1	10852	149.96	ND	5	0	24.26	0.00	12.13
**P02647|APOA1_HUMAN**	Apolipoprotein A-I OS=Homo sapiens GN=APOA1 PE=1 SV=1	30778	166.87	113.51	4	3	6.84	5.64	6.24
**P19823|ITIH2_HUMAN**	Inter-alpha-trypsin inhibitor heavy chain H2 OS=Homo sapiens GN=ITIH2 PE=1 SV=2	106463	166.91	185.28	6	9	2.97	4.89	3.93
**P01024|CO3_HUMAN**	Complement C3 OS=Homo sapiens GN=C3 PE=1 SV=2	187147	371.1	316.94	11	11	3.10	3.40	3.25
**P01857|IGHG1_HUMAN**	Ig gamma-1 chain C region OS=Homo sapiens GN=IGHG1 PE=1 SV=1	36106	76.6	ND	3	0	4.38	0.00	2.19
**P04114|APOB_HUMAN**	Apolipoprotein B-100 OS=Homo sapiens GN=APOB PE=1 SV=2	515611	294.32	264.52	19	18	1.94	2.02	1.98
**P02649|APOE_HUMAN**	Apolipoprotein E OS=Homo sapiens GN=APOE PE=1 SV=1	36154	ND	108.74	0	2	0.00	3.20	1.60
**P04004|VTNC_HUMAN**	Vitronectin OS=Homo sapiens GN=VTN PE=1 SV=1	54306	ND	121.91	0	3	0.00	3.20	1.60
**P01042|KNG1_HUMAN**	Kininogen-1 OS=Homo sapiens GN=KNG1 PE=1 SV=2	71957	58.71	44.31	2	1	1.46	0.80	1.13
**P09871|C1S_HUMAN**	Complement C1s subcomponent OS=Homo sapiens GN=C1S PE=1 SV=1	76685	164.73	ND	3	0	2.06	0.00	1.03
**P03952|KLKB1_HUMAN**	Plasma kallikrein OS=Homo sapiens GN=KLKB1 PE=1 SV=1	71370	ND	83.8	0	2	0.00	1.62	0.81
**O15370|SOX12_HUMAN**	Transcription factor SOX-12 OS=Homo sapiens GN=SOX12 PE=2 SV=2	34122	28.49	ND	1	0	1.54	0.00	0.77
**P02749|APOH_HUMAN**	Beta-2-glycoprotein 1 OS=Homo sapiens GN=APOH PE=1 SV=3	38298	ND	66.74	0	1	0.00	1.51	0.76
**P02765|FETUA_HUMAN**	Alpha-2-HS-glycoprotein OS=Homo sapiens GN=AHSG PE=1 SV=1	39325	ND	74.34	0	1	0.00	1.47	0.74
**P02760|AMBP_HUMAN**	Protein AMBP OS=Homo sapiens GN=AMBP PE=1 SV=1	39000	48.48	ND	1	0	1.35	0.00	0.68
**P10909|CLUS_HUMAN**	Clusterin OS=Homo sapiens GN=CLU PE=1 SV=1	52495	65.35	ND	1	0	1.00	0.00	0.50
**Q04756|HGFA_HUMAN**	Hepatocyte growth factor activator OS=Homo sapiens GN=HGFAC PE=1 SV=1	70682	ND	114.04	0	1	0.00	0.82	0.41
**P04003|C4BPA_HUMAN**	C4b-binding protein alpha chain OS=Homo sapiens GN=C4BPA PE=1 SV=2	67033	54.37	ND	1	0	0.79	0.00	0.39
**P00736|C1R_HUMAN**	Complement C1r subcomponent OS=Homo sapiens GN=C1R PE=1 SV=2	80119	ND	90.29	0	1	0.00	0.72	0.36
**P02787|TRFE_HUMAN**	Serotransferrin OS=Homo sapiens GN=TF PE=1 SV=3	77064	73.5	ND	1	0	0.68	0.00	0.34
**P00747|PLMN_HUMAN**	Plasminogen OS=Homo sapiens GN=PLG PE=1 SV=2	90569	47.46	ND	1	0	0.58	0.00	0.29

**P00450|CERU_HUMAN**	Ceruloplasmin OS=Homo sapiens GN=CP PE=1 SV=1	122205	ND	106.91	0	1	0.00	0.47	0.24
**Q9C0B9|ZCHC2_HUMAN**	Zinc finger CCHC domain-containing protein 2 OS=Homo sapiens GN=ZCCHC2 PE=1 SV=6	125936	25.76	ND	1	0	0.42	0.00	0.21
**Q92547|TOPB1_HUMAN**	DNA topoisomerase 2-binding protein 1 OS=Homo sapiens GN=TOPBP1 PE=1 SV=3	170678	26.34	ND	1	0	0.31	0.00	0.15
**P0C0L4|CO4A_HUMAN**	Complement C4-A OS=Homo sapiens GN=C4A PE=1 SV=1	192770	26.38	ND	1	0	0.27	0.00	0.14
**P0C0L5|CO4B_HUMAN**	Complement C4-B OS=Homo sapiens GN=C4B PE=1 SV=1	192792	26.38	ND	1	0	0.27	0.00	0.14
**Q8NEY1|NAV1_HUMAN**	Neuron navigator 1 OS=Homo sapiens GN=NAV1 PE=1 SV=2	202470	25.68	ND	1	0	0.26	0.00	0.13
**Q8WXI7|MUC16_HUMAN**	Mucin-16 OS=Homo sapiens GN=MUC16 PE=1 SV=2	2358133	27.44	ND	1	0	0.02	0.00	0.01

Table S1f: (12% EMT + 100% human plasma)

Accession	Description	Mw	-10lgP		Spectra		NpSpCk		Average NpSpCk
			S 1	S 2	S 1	S 2	S 1	S 2	
**P02656|APOC3_HUMAN**	Apolipoprotein C-III OS=Homo sapiens GN=APOC3 PE=1 SV=1	10852	189.29	177.2	5	6	27.59	25.75	26.67
**P02671|FIBA_HUMAN**	Fibrinogen alpha chain OS=Homo sapiens GN=FGA PE=1 SV=2	94973	414.34	334.15	29	29	18.28	14.22	16.25
**P02679|FIBG_HUMAN**	Fibrinogen gamma chain OS=Homo sapiens GN=FGG PE=1 SV=3	51512	314.11	283.84	13	14	15.11	12.66	13.88
**P02675|FIBB_HUMAN**	Fibrinogen beta chain OS=Homo sapiens GN=FGB PE=1 SV=2	55928	284.67	200.7	9	13	9.64	10.83	10.23
**P02768|ALBU_HUMAN**	Serum albumin OS=Homo sapiens GN=ALB PE=1 SV=2	69367	326.93	334.97	11	16	9.50	10.74	10.12
**P02647|APOA1_HUMAN**	Apolipoprotein A-I OS=Homo sapiens GN=APOA1 PE=1 SV=1	30778	160.72	166.71	2	5	3.89	7.57	5.73
**P19823|ITIH2_HUMAN**	Inter-alpha-trypsin inhibitor heavy chain H2 OS=Homo sapiens GN=ITIH2 PE=1 SV=2	106463	130.81	229.14	7	8	3.94	3.50	3.72
**P04004|VTNC_HUMAN**	Vitronectin OS=Homo sapiens GN=VTN PE=1 SV=1	54306	198.44	111.32	3	3	3.31	2.57	2.94
**P01024|CO3_HUMAN**	Complement C3 OS=Homo sapiens GN=C3 PE=1 SV=2	187147	373.99	365.84	9	11	2.88	2.74	2.81
**P04003|C4BPA_HUMAN**	C4b-binding protein alpha chain OS=Homo sapiens GN=C4BPA PE=1 SV=2	67033	92.93	53.7	2	2	1.79	1.39	1.59
**P01857|IGHG1_HUMAN**	Ig gamma-1 chain C region OS=Homo sapiens GN=IGHG1 PE=1 SV=1	36106	ND	96.97	0	2	0.00	2.58	1.29
**P04114|APOB_HUMAN**	Apolipoprotein B-100 OS=Homo sapiens GN=APOB PE=1 SV=2	515611	238.42	208.01	10	9	1.16	0.81	0.99
**P02748|CO9_HUMAN**	Complement component C9 OS=Homo sapiens GN=C9 PE=1 SV=2	63173	ND	62.79	0	2	0.00	1.47	0.74
**P00751|CFAB_HUMAN**	Complement factor B OS=Homo sapiens GN=CFB PE=1 SV=2	85533	45.2	ND	2	0	1.40	0.00	0.70
**P02787|TRFE_HUMAN**	Serotransferrin OS=Homo sapiens GN=TF PE=1 SV=3	77064	36.21	95.17	1	1	0.78	0.60	0.69
**P02749|APOH_HUMAN**	Beta-2-glycoprotein 1 OS=Homo sapiens GN=APOH PE=1 SV=3	38298	ND	55.03	0	1	0.00	1.22	0.61
**P00736|C1R_HUMAN**	Complement C1r subcomponent OS=Homo sapiens GN=C1R PE=1 SV=2	80119	74.95	ND	1	0	0.75	0.00	0.37
**P07358|CO8B_HUMAN**	Complement component C8 beta chain OS=Homo sapiens GN=C8B PE=1 SV=3	67047	ND	76.88	0	1	0.00	0.69	0.35
**P01042|KNG1_HUMAN**	Kininogen-1 OS=Homo sapiens GN=KNG1 PE=1 SV=2	71957	ND	44.95	0	1	0.00	0.65	0.32

Table S2 (a-f): nLC-MS/MS combined with PEAKS DB analysis of the corona (protein content) of Nano-sized FAU zeolite NPs. The accession number, gene name, species (Human), protein description, identification score (-10lgP), molecular wight (Mw) in kDa, total spectra per protein of EMT (4%, 8% and 12%) zeolite NPs incubated with human plasma (10%, and 100%), together with their relative amount (NpSpCk value).Sample (S).

Table S2a: (4% FAU + 10% human plasma)

Accession	Description	Mw	-10lgP S 1	-10lgP S 2	Spectra S 1	Spectra S 2	NpSpCk S 1	NpSpCk S 2	Average NpSpCk
**P01857|IGHG1_HUMAN**	Ig gamma-1 chain C region OS=Homo sapiens GN=IGHG1 PE=1 SV=1	36106	402.73	349.27	35	34	11.38	18.43	14.90
**P02768|ALBU_HUMAN**	Serum albumin OS=Homo sapiens GN=ALB PE=1 SV=2	69367	424.02	378.19	54	43	9.14	12.13	10.63
**P02675|FIBB_HUMAN**	Fibrinogen beta chain OS=Homo sapiens GN=FGB PE=1 SV=2	55928	445.93	279.25	36	17	7.56	5.95	6.75
**P02679|FIBG_HUMAN**	Fibrinogen gamma chain OS=Homo sapiens GN=FGG PE=1 SV=3	51512	374.27	302.79	29	18	6.61	6.84	6.72
**P01859|IGHG2_HUMAN**	Ig gamma-2 chain C region OS=Homo sapiens GN=IGHG2 PE=1 SV=2	35901	259.37	236.08	10	12	3.27	6.54	4.91
**P01024|CO3_HUMAN**	Complement C3 OS=Homo sapiens GN=C3 PE=1 SV=2	187147	505.76	459.24	48	40	3.01	4.18	3.60
**P02656|APOC3_HUMAN**	Apolipoprotein C-III OS=Homo sapiens GN=APOC3 PE=1 SV=1	10852	147.88	135.2	3	2	3.25	3.61	3.43
**P04196|HRG_HUMAN**	Histidine-rich glycoprotein OS=Homo sapiens GN=HRG PE=1 SV=1	59578	211.71	254.3	11	13	2.17	4.27	3.22
**P02647|APOA1_HUMAN**	Apolipoprotein A-I OS=Homo sapiens GN=APOA1 PE=1 SV=1	30778	211.05	220.47	6	6	2.29	3.81	3.05
**P02671|FIBA_HUMAN**	Fibrinogen alpha chain OS=Homo sapiens GN=FGA PE=1 SV=2	94973	321.06	210.34	25	12	3.09	2.47	2.78
**P0CG06|LAC3_HUMAN**	Ig lambda-3 chain C regions OS=Homo sapiens GN=IGLC3 PE=1 SV=1	11237	168.84	99.04	2	1	2.09	1.74	1.92
**P0CF74|LAC6_HUMAN**	Ig lambda-6 chain C region OS=Homo sapiens GN=IGLC6 PE=4 SV=1	11277	168.84	99.04	2	1	2.08	1.74	1.91
**P0CG05|LAC2_HUMAN**	Ig lambda-2 chain C regions OS=Homo sapiens GN=IGLC2 PE=1 SV=1	11294	168.84	99.04	2	1	2.08	1.73	1.91
**A0M8Q6|LAC7_HUMAN**	Ig lambda-7 chain C region OS=Homo sapiens GN=IGLC7 PE=1 SV=2	11303	168.84	99.04	2	1	2.08	1.73	1.90
**P0C0L4|CO4A_HUMAN**	Complement C4-A OS=Homo sapiens GN=C4A PE=1 SV=1	192770	371.39	339.67	25	19	1.52	1.93	1.73
**P0C0L5|CO4B_HUMAN**	Complement C4-B OS=Homo sapiens GN=C4B PE=1 SV=1	192792	371.39	339.67	25	19	1.52	1.93	1.73
**P01622|KV304_HUMAN**	Ig kappa chain V-III region Ti OS=Homo sapiens PE=1 SV=1	11788	185.37	ND	3	0	2.99	0.00	1.49
**P04114|APOB_HUMAN**	Apolipoprotein B-100 OS=Homo sapiens GN=APOB PE=1 SV=2	515611	437.14	410.42	50	46	1.14	1.75	1.44

**P19823|ITIH2_HUMAN**	Inter-alpha-trypsin inhibitor heavy chain H2 OS=Homo sapiens GN=ITIH2 PE=1 SV=2	106463	168.6	224.72	8	10	0.88	1.84	1.36
**P07360|CO8G_HUMAN**	Complement component C8 gamma chain OS=Homo sapiens GN=C8G PE=1 SV=3	22277	217.82	ND	5	0	2.63	0.00	1.32
**P36955|PEDF_HUMAN**	Pigment epithelium-derived factor OS=Homo sapiens GN=SERPINF1 PE=1 SV=4	46312	107.9	161.18	3	4	0.76	1.69	1.23
**P19827|ITIH1_HUMAN**	Inter-alpha-trypsin inhibitor heavy chain H1 OS=Homo sapiens GN=ITIH1 PE=1 SV=3	101389	252.18	258.85	6	8	0.69	1.54	1.12
**P04004|VTNC_HUMAN**	Vitronectin OS=Homo sapiens GN=VTN PE=1 SV=1	54306	198.24	141.01	7	2	1.51	0.72	1.12
**P01623|KV305_HUMAN**	Ig kappa chain V-III region WOL OS=Homo sapiens PE=1 SV=1	11746	185.37	ND	2	0	2.00	0.00	1.00
**P01620|KV302_HUMAN**	Ig kappa chain V-III region SIE OS=Homo sapiens PE=1 SV=1	11775	185.37	ND	2	0	1.99	0.00	1.00
**P04206|KV307_HUMAN**	Ig kappa chain V-III region GOL OS=Homo sapiens PE=1 SV=1	11830	185.37	ND	2	0	1.98	0.00	0.99
**P00747|PLMN_HUMAN**	Plasminogen OS=Homo sapiens GN=PLG PE=1 SV=2	90569	179.08	135.81	7	4	0.91	0.86	0.89
**P04003|C4BPA_HUMAN**	C4b-binding protein alpha chain OS=Homo sapiens GN=C4BPA PE=1 SV=2	67033	130.88	132.91	5	3	0.88	0.88	0.88
**P0CG04|LAC1_HUMAN**	Ig lambda-1 chain C regions OS=Homo sapiens GN=IGLC1 PE=1 SV=1	11348	ND	99.04	0	1	0.00	1.72	0.86
**P01834|IGKC_HUMAN**	Ig kappa chain C region OS=Homo sapiens GN=IGKC PE=1 SV=1	11609	ND	109.16	0	1	0.00	1.69	0.84
**P18135|KV312_HUMAN**	Ig kappa chain V-III region HAH OS=Homo sapiens PE=2 SV=1	14073	185.37	ND	2	0	1.67	0.00	0.83
**P18136|KV313_HUMAN**	Ig kappa chain V-III region HIC OS=Homo sapiens PE=2 SV=2	14089	185.37	ND	2	0	1.67	0.00	0.83
**P08603|CFAH_HUMAN**	Complement factor H OS=Homo sapiens GN=CFH PE=1 SV=4	139096	139.72	141.55	6	5	0.51	0.70	0.60
**P02748|CO9_HUMAN**	Complement component C9 OS=Homo sapiens GN=C9 PE=1 SV=2	63173	98.18	54.15	3	2	0.56	0.62	0.59
**P35858|ALS_HUMAN**	Insulin-like growth factor-binding protein complex acid labile subunit OS=Homo sapiens GN=IGFALS PE=1 SV=1	66035	65.51	100.21	1	3	0.18	0.89	0.53
**P01042|KNG1_HUMAN**	Kininogen-1 OS=Homo sapiens GN=KNG1 PE=1 SV=2	71957	182.82	95.03	3	2	0.49	0.54	0.52
**P00751|CFAB_HUMAN**	Complement factor B OS=Homo sapiens GN=CFB PE=1 SV=2	85533	175.93	147.96	4	2	0.55	0.46	0.50
**P01596|KV104_HUMAN**	Ig kappa chain V-I region CAR OS=Homo sapiens PE=1 SV=1	11704	110.99	ND	1	0	1.00	0.00	0.50
**P01609|KV117_HUMAN**	Ig kappa chain V-I region Scw OS=Homo sapiens PE=1 SV=1	11764	32.75	ND	1	0	1.00	0.00	0.50
**P01598|KV106_HUMAN**	Ig kappa chain V-I region EU OS=Homo sapiens PE=1 SV=1	11788	110.99	ND	1	0	1.00	0.00	0.50
**P01593|KV101_HUMAN**	Ig kappa chain V-I region AG OS=Homo sapiens PE=1 SV=1	11992	32.75	ND	1	0	0.98	0.00	0.49
**P02649|APOE_HUMAN**	Apolipoprotein E OS=Homo sapiens GN=APOE PE=1 SV=1	36154	182.53	ND	3	0	0.97	0.00	0.49
**P02749|APOH_HUMAN**	Beta-2-glycoprotein 1 OS=Homo sapiens GN=APOH PE=1 SV=3	38298	122.67	ND	3	0	0.92	0.00	0.46
**P01602|KV110_HUMAN**	Ig kappa chain V-I region HK102 (Fragment) OS=Homo sapiens	12768	110.99	ND	1	0	0.92	0.00	0.46

	GN=IGKV1-5 PE=4 SV=1								
**P01777|HV316_HUMAN**	Ig heavy chain V-III region TEI OS=Homo sapiens PE=1 SV=1	12802	114.42	ND	1	0	0.92	0.00	0.46
**P01766|HV305_HUMAN**	Ig heavy chain V-III region BRO OS=Homo sapiens PE=1 SV=1	13227	114.42	ND	1	0	0.89	0.00	0.44
**P02746|C1QB_HUMAN**	Complement C1q subcomponent subunit B OS=Homo sapiens GN=C1QB PE=1 SV=3	26722	96.79	ND	2	0	0.88	0.00	0.44
**B9A064|IGLL5_HUMAN**	Immunoglobulin lambda-like polypeptide 5 OS=Homo sapiens GN=IGLL5 PE=2 SV=2	23063	ND	99.04	0	1	0.00	0.85	0.42
**P09871|C1S_HUMAN**	Complement C1s subcomponent OS=Homo sapiens GN=C1S PE=1 SV=1	76685	89.6	53.89	3	1	0.46	0.26	0.36
**P06681|CO2_HUMAN**	Complement C2 OS=Homo sapiens GN=C2 PE=1 SV=2	83268	ND	69.38	0	3	0.00	0.71	0.35
**P07358|CO8B_HUMAN**	Complement component C8 beta chain OS=Homo sapiens GN=C8B PE=1 SV=3	67047	123.03	66.73	2	1	0.35	0.29	0.32
**P02751|FINC_HUMAN**	Fibronectin OS=Homo sapiens GN=FN1 PE=1 SV=4	262622	294.36	174.38	8	3	0.36	0.22	0.29
**P00738|HPT_HUMAN**	Haptoglobin OS=Homo sapiens GN=HP PE=1 SV=1	45205	124.32	ND	2	0	0.52	0.00	0.26
**P02760|AMBP_HUMAN**	Protein AMBP OS=Homo sapiens GN=AMBP PE=1 SV=1	39000	ND	96.36	0	1	0.00	0.50	0.25
**Q96IY4|CBPB2_HUMAN**	Carboxypeptidase B2 OS=Homo sapiens GN=CPB2 PE=1 SV=2	48424	61.73	ND	2	0	0.48	0.00	0.24
**P04220|MUCB_HUMAN**	Ig mu heavy chain disease protein OS=Homo sapiens PE=1 SV=1	43057	ND	68.72	0	1	0.00	0.45	0.23
**P03952|KLKB1_HUMAN**	Plasma kallikrein OS=Homo sapiens GN=KLKB1 PE=1 SV=1	71370	86.73	62.71	1	1	0.16	0.27	0.22
**P01031|CO5_HUMAN**	Complement C5 OS=Homo sapiens GN=C5 PE=1 SV=4	188304	ND	77.96	0	4	0.00	0.42	0.21
**P01876|IGHA1_HUMAN**	Ig alpha-1 chain C region OS=Homo sapiens GN=IGHA1 PE=1 SV=2	37655	85.66	ND	1	0	0.31	0.00	0.16
**O00311|CDC7_HUMAN**	Cell division cycle 7-related protein kinase OS=Homo sapiens GN=CDC7 PE=1 SV=1	63888	ND	26.15	0	1	0.00	0.31	0.15
**P00748|FA12_HUMAN**	Coagulation factor XII OS=Homo sapiens GN=F12 PE=1 SV=3	67792	ND	33.98	0	1	0.00	0.29	0.14
**P10909|CLUS_HUMAN**	Clusterin OS=Homo sapiens GN=CLU PE=1 SV=1	52495	36.94	ND	1	0	0.22	0.00	0.11
**P10643|CO7_HUMAN**	Complement component C7 OS=Homo sapiens GN=C7 PE=1 SV=2	93518	ND	115.11	0	1	0.00	0.21	0.10
**P00450|CERU_HUMAN**	Ceruloplasmin OS=Homo sapiens GN=CP PE=1 SV=1	122205	ND	32.7	0	1	0.00	0.16	0.08
**P02787|TRFE_HUMAN**	Serotransferrin OS=Homo sapiens GN=TF PE=1 SV=3	77064	29.77	ND	1	0	0.15	0.00	0.08
**P06396|GELS_HUMAN**	Gelsolin OS=Homo sapiens GN=GSN PE=1 SV=1	85697	41.76	ND	1	0	0.14	0.00	0.07
**Q5JPB2|ZN831_HUMAN**	Zinc finger protein 831 OS=Homo sapiens GN=ZNF831 PE=2 SV=4	177948	35.79	ND	2	0	0.13	0.00	0.07
**P09327|VILI_HUMAN**	Villin-1 OS=Homo sapiens GN=VIL1 PE=1 SV=4	92695	28.63	ND	1	0	0.13	0.00	0.06
**P12259|FA5_HUMAN**	Coagulation factor V OS=Homo sapiens GN=F5 PE=1 SV=4	251701	ND	27.38	0	1	0.00	0.08	0.04

**Q09666IAHNK_HUMAN**	Neuroblast differentiation-associated protein AHNAK OS=Homo sapiens GN=AHNAK PE=1SV=2	629114	ND	27.22	0	1	0.00	0.03	0.02
**Q8WZ42ITITIN_HUMAN**	Titin OS=Homo sapiens GN=TTN PE=1SV=4	3815922	ND	26.21	0	5	0.00	0.03	0.01

Table S2b: (8% FAU + 10% human plasma)

Accession	Description	Mw	-10lgP		Spectra		NpSpCk		Average NpSpCk
			S 1	S 2	S 1	S 2	S 1	S 2	
**P02768|ALBU_HUMAN**	Serum albumin OS=Homo sapiens GN=ALB PE=1 SV=2	69367	385.7	398.94	51	31	14.25	19.50	16.87
**P01857|IGHG1_HUMAN**	Ig gamma-1 chain C region OS=Homo sapiens GN=IGHG1 PE=1 SV=1	36106	331.02	316.43	23	13	12.35	15.71	14.03
**P02675|FIBB_HUMAN**	Fibrinogen beta chain OS=Homo sapiens GN=FGB PE=1 SV=2	55928	476.1	255.78	28	11	9.70	8.58	9.14
**P02679|FIBG_HUMAN**	Fibrinogen gamma chain OS=Homo sapiens GN=FGG PE=1 SV=3	51512	372	321.67	20	12	7.52	10.17	8.84
**P01024|CO3_HUMAN**	Complement C3 OS=Homo sapiens GN=C3 PE=1 SV=2	187147	522.66	508.2	44	28	4.56	6.53	5.54
**P02671|FIBA_HUMAN**	Fibrinogen alpha chain OS=Homo sapiens GN=FGA PE=1 SV=2	94973	323.61	288.03	21	10	4.29	4.59	4.44
**P0C0L4|CO4A_HUMAN**	Complement C4-A OS=Homo sapiens GN=C4A PE=1 SV=1	192770	338.37	346.73	32	19	3.22	4.30	3.76
**P0C0L5|CO4B_HUMAN**	Complement C4-B OS=Homo sapiens GN=C4B PE=1 SV=1	192792	338.37	346.73	32	19	3.22	4.30	3.76
**P02647|APOA1_HUMAN**	Apolipoprotein A-I OS=Homo sapiens GN=APOA1 PE=1 SV=1	30778	166.48	86.43	7	2	4.41	2.84	3.62
**P01859|IGHG2_HUMAN**	Ig gamma-2 chain C region OS=Homo sapiens GN=IGHG2 PE=1 SV=2	35901	188.05	108.71	6	3	3.24	3.65	3.44
**P02649|APOE_HUMAN**	Apolipoprotein E OS=Homo sapiens GN=APOE PE=1 SV=1	36154	ND	98.31	0	4	0.00	4.83	2.41
**P19823|ITIH2_HUMAN**	Inter-alpha-trypsin inhibitor heavy chain H2 OS=Homo sapiens GN=ITIH2 PE=1 SV=2	106463	248.34	242.24	8	8	1.46	3.28	2.37
**P02654|APOC1_HUMAN**	Apolipoprotein C-I OS=Homo sapiens GN=APOC1 PE=1 SV=1	9332	39.77	ND	2	0	4.15	0.00	2.08
**P04114|APOB_HUMAN**	Apolipoprotein B-100 OS=Homo sapiens GN=APOB PE=1 SV=2	515611	433.27	478.73	38	32	1.43	2.71	2.07
**P19827|ITIH1_HUMAN**	Inter-alpha-trypsin inhibitor heavy chain H1 OS=Homo sapiens GN=ITIH1 PE=1 SV=3	101389	274.91	120.79	7	4	1.34	1.72	1.53
**P00747|PLMN_HUMAN**	Plasminogen OS=Homo sapiens GN=PLG PE=1 SV=2	90569	192.07	156.33	5	4	1.07	1.93	1.50
**P04004|VTNC_HUMAN**	Vitronectin OS=Homo sapiens GN=VTN PE=1 SV=1	54306	108.21	80.7	3	2	1.07	1.61	1.34
**P36955|PEDF_HUMAN**	Pigment epithelium-derived factor OS=Homo sapiens GN=SERPINF1 PE=1 SV=4	46312	71.36	48.83	2	1	0.84	0.94	0.89
**P01600|KV108_HUMAN**	Ig kappa chain V-I region Hau OS=Homo sapiens PE=1 SV=1	11671	124.56	ND	1	0	1.66	0.00	0.83
**P80362|KV125_HUMAN**	Ig kappa chain V-I region WAT OS=Homo sapiens PE=1 SV=1	11737	124.56	ND	1	0	1.65	0.00	0.83
**P01609|KV117_HUMAN**	Ig kappa chain V-I region Scw OS=Homo sapiens PE=1 SV=1	11764	124.56	ND	1	0	1.65	0.00	0.82
**P01608|KV116_HUMAN**	Ig kappa chain V-I region Roy OS=Homo sapiens PE=1 SV=1	11782	124.56	ND	1	0	1.64	0.00	0.82

**P01599|KV107_HUMAN**	Ig kappa chain V-I region Gal OS=Homo sapiens PE=1 SV=1	11814	124.56	ND	1	0	1.64	0.00	0.82
**P01610|KV118_HUMAN**	Ig kappa chain V-I region WEA OS=Homo sapiens PE=1 SV=1	11840	124.56	ND	1	0	1.64	0.00	0.82
**P01607|KV115_HUMAN**	Ig kappa chain V-I region Rei OS=Homo sapiens PE=1 SV=1	11902	124.56	ND	1	0	1.63	0.00	0.81
**P01594|KV102_HUMAN**	Ig kappa chain V-I region AU OS=Homo sapiens PE=1 SV=1	11939	124.56	ND	1	0	1.62	0.00	0.81
**P01593|KV101_HUMAN**	Ig kappa chain V-I region AG OS=Homo sapiens PE=1 SV=1	11992	124.56	ND	1	0	1.62	0.00	0.81
**P01601|KV109_HUMAN**	Ig kappa chain V-I region HK101 (Fragment) OS=Homo sapiens PE=4 SV=1	12800	124.56	ND	1	0	1.51	0.00	0.76
**P04431|KV123_HUMAN**	Ig kappa chain V-I region Walker OS=Homo sapiens PE=4 SV=1	14069	124.56	ND	1	0	1.38	0.00	0.69
**P04432|KV124_HUMAN**	Ig kappa chain V-I region Daudi OS=Homo sapiens PE=4 SV=1	14235	124.56	ND	1	0	1.36	0.00	0.68
**P00751|CFAB_HUMAN**	Complement factor B OS=Homo sapiens GN=CFB PE=1 SV=2	85533	ND	68.49	0	2	0.00	1.02	0.51
**P13671|CO6_HUMAN**	Complement component C6 OS=Homo sapiens GN=C6 PE=1 SV=3	104786	71.26	139.61	2	1	0.37	0.42	0.39
**P04196|HRG_HUMAN**	Histidine-rich glycoprotein OS=Homo sapiens GN=HRG PE=1 SV=1	59578	ND	117.59	0	1	0.00	0.73	0.37
**P07358|CO8B_HUMAN**	Complement component C8 beta chain OS=Homo sapiens GN=C8B PE=1 SV=3	67047	ND	76.76	0	1	0.00	0.65	0.33
**P02748|CO9_HUMAN**	Complement component C9 OS=Homo sapiens GN=C9 PE=1 SV=2	63173	101.25	ND	2	0	0.61	0.00	0.31
**P35858|ALS_HUMAN**	Insulin-like growth factor-binding protein complex acid labile subunit OS=Homo sapiens GN=IGFALS PE=1 SV=1	66035	135.1	ND	2	0	0.59	0.00	0.29
**P08603|CFAH_HUMAN**	Complement factor H OS=Homo sapiens GN=CFH PE=1 SV=4	139096	144.99	ND	4	0	0.56	0.00	0.28
**Q14624|ITIH4_HUMAN**	Inter-alpha-trypsin inhibitor heavy chain H4 OS=Homo sapiens GN=ITIH4 PE=1 SV=4	103357	108.47	ND	2	0	0.38	0.00	0.19
**P02751|FINC_HUMAN**	Fibronectin OS=Homo sapiens GN=FN1 PE=1 SV=4	262622	173.45	ND	4	0	0.30	0.00	0.15
**P01031|CO5_HUMAN**	Complement C5 OS=Homo sapiens GN=C5 PE=1 SV=4	188304	36.1	ND	1	0	0.10	0.00	0.05

Table S2c: (12% FAU + 10% human plasma)

Accession	Description	Mw	-10lgP S 1	-10lgP S 2	Spectra S 1	Spectra S 2	NpSpCk S 1	NpSpCk S 2	Average NpSpCk
**P02768|ALBU_HUMAN**	Serum albumin OS=Homo sapiens GN=ALB PE=1 SV=2	69367	451.51	465.11	70	50	14.12	16.34	15.23
**P01857|IGHG1_HUMAN**	Ig gamma-1 chain C region OS=Homo sapiens GN=IGHG1 PE=1 SV=1	36106	346.79	346.44	29	24	11.24	15.07	13.15
**P02679|FIBG_HUMAN**	Fibrinogen gamma chain OS=Homo sapiens GN=FGG PE=1 SV=3	51512	398.18	318.82	29	17	7.88	7.48	7.68
**P02675|FIBB_HUMAN**	Fibrinogen beta chain OS=Homo sapiens GN=FGB PE=1 SV=2	55928	422.76	289.12	33	14	8.25	5.67	6.96
**P01861|IGHG4_HUMAN**	Ig gamma-4 chain C region OS=Homo sapiens GN=IGHG4 PE=1 SV=1	35941	ND	355.35	0	22	0.00	13.88	6.94
**P01024|CO3_HUMAN**	Complement C3 OS=Homo sapiens GN=C3 PE=1 SV=2	187147	515.48	536.65	45	43	3.36	5.21	4.29
**P01859|IGHG2_HUMAN**	Ig gamma-2 chain C region OS=Homo sapiens GN=IGHG2 PE=1 SV=2	35901	270.62	208.53	10	5	3.90	3.16	3.53
**P02671|FIBA_HUMAN**	Fibrinogen alpha chain OS=Homo sapiens GN=FGA PE=1 SV=2	94973	351.5	278.85	26	11	3.83	2.63	3.23
**P02656|APOC3_HUMAN**	Apolipoprotein C-III OS=Homo sapiens GN=APOC3 PE=1 SV=1	10852	ND	142.94	0	3	0.00	6.27	3.13
**P02647|APOA1_HUMAN**	Apolipoprotein A-I OS=Homo sapiens GN=APOA1 PE=1 SV=1	30778	216.29	209.03	5	5	2.27	3.68	2.98
**P04196|HRG_HUMAN**	Histidine-rich glycoprotein OS=Homo sapiens GN=HRG PE=1 SV=1	59578	193.9	241.3	6	10	1.41	3.80	2.61
**P0C0L4|CO4A_HUMAN**	Complement C4-A OS=Homo sapiens GN=C4A PE=1 SV=1	192770	360.85	373.7	26	19	1.89	2.23	2.06
**P0C0L5|CO4B_HUMAN**	Complement C4-B OS=Homo sapiens GN=C4B PE=1 SV=1	192792	360.85	373.7	26	19	1.89	2.23	2.06
**P04114|APOB_HUMAN**	Apolipoprotein B-100 OS=Homo sapiens GN=APOB PE=1 SV=2	515611	437.62	396.75	40	33	1.09	1.45	1.27
**P07360|CO8G_HUMAN**	Complement component C8 gamma chain OS=Homo sapiens GN=C8G PE=1 SV=3	22277	200.36	ND	4	0	2.51	0.00	1.26
**P0CG06|LAC3_HUMAN**	Ig lambda-3 chain C regions OS=Homo sapiens GN=IGLC3 PE=1 SV=1	11237	156.62	ND	2	0	2.49	0.00	1.24
**P0CF74|LAC6_HUMAN**	Ig lambda-6 chain C region OS=Homo sapiens GN=IGLC6 PE=4 SV=1	11277	156.62	ND	2	0	2.48	0.00	1.24
**P0CG05|LAC2_HUMAN**	Ig lambda-2 chain C regions OS=Homo sapiens GN=IGLC2 PE=1 SV=1	11294	156.62	ND	2	0	2.48	0.00	1.24
**A0M8Q6|LAC7_HUMAN**	Ig lambda-7 chain C region OS=Homo sapiens GN=IGLC7 PE=1 SV=2	11303	156.62	ND	2	0	2.48	0.00	1.24
**P02649|APOE_HUMAN**	Apolipoprotein E OS=Homo sapiens GN=APOE PE=1 SV=1	36154	205.82	54.13	4	1	1.55	0.63	1.09
**P19827|ITIH1_HUMAN**	Inter-alpha-trypsin inhibitor heavy chain H1 OS=Homo sapiens GN=ITIH1 PE=1 SV=3	101389	307.15	289.47	7	5	0.97	1.12	1.04

**P36955|PEDF_HUMAN**	Pigment epithelium-derived factor OS=Homo sapiens GN=SERPINF1 PE=1 SV=4	46312	138.82	113.03	3	2	0.91	0.98	0.94
**P04004|VTNC_HUMAN**	Vitronectin OS=Homo sapiens GN=VTN PE=1 SV=1	54306	180.45	138.35	4	2	1.03	0.83	0.93
**P00747|PLMN_HUMAN**	Plasminogen OS=Homo sapiens GN=PLG PE=1 SV=2	90569	175.26	165.37	5	4	0.77	1.00	0.89
**P19823|ITIH2_HUMAN**	Inter-alpha-trypsin inhibitor heavy chain H2 OS=Homo sapiens GN=ITIH2 PE=1 SV=2	106463	216.1	106.59	6	4	0.79	0.85	0.82
**P01621|KV303_HUMAN**	Ig kappa chain V-III region NG9 (Fragment) OS=Homo sapiens PE=1 SV=1	10729	114.74	ND	1	0	1.30	0.00	0.65
**P04003|C4BPA_HUMAN**	C4b-binding protein alpha chain OS=Homo sapiens GN=C4BPA PE=1 SV=2	67033	111.26	89.02	3	2	0.63	0.68	0.65
**P01834|IGKC_HUMAN**	Ig kappa chain C region OS=Homo sapiens GN=IGKC PE=1 SV=1	11609	104.82	ND	1	0	1.21	0.00	0.60
**P01623|KV305_HUMAN**	Ig kappa chain V-III region WOL OS=Homo sapiens PE=1 SV=1	11746	114.74	ND	1	0	1.19	0.00	0.60
**P01620|KV302_HUMAN**	Ig kappa chain V-III region SIE OS=Homo sapiens PE=1 SV=1	11775	114.74	ND	1	0	1.19	0.00	0.59
**P01622|KV304_HUMAN**	Ig kappa chain V-III region Ti OS=Homo sapiens PE=1 SV=1	11788	114.74	ND	1	0	1.19	0.00	0.59
**P04206|KV307_HUMAN**	Ig kappa chain V-III region GOL OS=Homo sapiens PE=1 SV=1	11830	114.74	ND	1	0	1.18	0.00	0.59
**P01042|KNG1_HUMAN**	Kininogen-1 OS=Homo sapiens GN=KNG1 PE=1 SV=2	71957	154.91	76.12	4	1	0.78	0.32	0.55
**P02746|C1QB_HUMAN**	Complement C1q subcomponent subunit B OS=Homo sapiens GN=C1QB PE=1 SV=3	26722	93.18	ND	2	0	1.05	0.00	0.52
**P08603|CFAH_HUMAN**	Complement factor H OS=Homo sapiens GN=CFH PE=1 SV=4	139096	168.18	99.2	7	2	0.70	0.33	0.51
**P18135|KV312_HUMAN**	Ig kappa chain V-III region HAH OS=Homo sapiens PE=2 SV=1	14073	114.74	ND	1	0	0.99	0.00	0.50
**P18136|KV313_HUMAN**	Ig kappa chain V-III region HIC OS=Homo sapiens PE=2 SV=2	14089	114.74	ND	1	0	0.99	0.00	0.50
**P04207|KV308_HUMAN**	Ig kappa chain V-III region CLL OS=Homo sapiens PE=1 SV=2	14275	95.87	ND	1	0	0.98	0.00	0.49
**P02760|AMBP_HUMAN**	Protein AMBP OS=Homo sapiens GN=AMBP PE=1 SV=1	39000	89.71	103.08	1	1	0.36	0.58	0.47
**P07358|CO8B_HUMAN**	Complement component C8 beta chain OS=Homo sapiens GN=C8B PE=1 SV=3	67047	76.87	136.66	1	2	0.21	0.68	0.44
**P02749|APOH_HUMAN**	Beta-2-glycoprotein 1 OS=Homo sapiens GN=APOH PE=1 SV=3	38298	97.84	ND	2	0	0.73	0.00	0.37
**P02751|FINC_HUMAN**	Fibronectin OS=Homo sapiens GN=FN1 PE=1 SV=4	262622	300.72	141.14	8	3	0.43	0.26	0.34
**P02748|CO9_HUMAN**	Complement component C9 OS=Homo sapiens GN=C9 PE=1 SV=2	63173	135.08	ND	3	0	0.66	0.00	0.33
**P04220|MUCB_HUMAN**	Ig mu heavy chain disease protein OS=Homo sapiens PE=1 SV=1	43057	93.91	ND	2	0	0.65	0.00	0.32
**P05452|TETN_HUMAN**	Tetranectin OS=Homo sapiens GN=CLEC3B PE=1 SV=3	22537	39.25	ND	1	0	0.62	0.00	0.31
**P06681|CO2_HUMAN**	Complement C2 OS=Homo sapiens GN=C2 PE=1 SV=2	83268	52	46.92	2	1	0.34	0.27	0.30
**Q96IY4|CBPB2_HUMAN**	Carboxypeptidase B2 OS=Homo sapiens GN=CPB2 PE=1 SV=2	48424	71.37	ND	2	0	0.58	0.00	0.29

**P01871|IGHM_HUMAN**	Ig mu chain C region OS=Homo sapiens GN=IGHM PE=1 SV=3	49307	93.91	ND	2	0	0.57	0.00	0.28
**P09871|C1S_HUMAN**	Complement C1s subcomponent OS=Homo sapiens GN=C1S PE=1 SV=1	76685	81.01	ND	3	0	0.55	0.00	0.27
**P00751|CFAB_HUMAN**	Complement factor B OS=Homo sapiens GN=CFB PE=1 SV=2	85533	ND	132.81	0	2	0.00	0.53	0.27
**E7ETH6|Z587B_HUMAN**	Zinc finger protein 587B OS=Homo sapiens GN=ZNF587B PE=2 SV=1	45541	ND	31.15	0	1	0.00	0.50	0.25
**P01031|CO5_HUMAN**	Complement C5 OS=Homo sapiens GN=C5 PE=1 SV=4	188304	ND	162.95	0	4	0.00	0.48	0.24
**P10909|CLUS_HUMAN**	Clusterin OS=Homo sapiens GN=CLU PE=1 SV=1	52495	ND	34.65	0	1	0.00	0.43	0.22
**P07357|CO8A_HUMAN**	Complement component C8 alpha chain OS=Homo sapiens GN=C8A PE=1 SV=2	65163	131.5	ND	2	0	0.43	0.00	0.21
**P01876|IGHA1_HUMAN**	Ig alpha-1 chain C region OS=Homo sapiens GN=IGHA1 PE=1 SV=2	37655	102.18	ND	1	0	0.37	0.00	0.19
**P10643|CO7_HUMAN**	Complement component C7 OS=Homo sapiens GN=C7 PE=1 SV=2	93518	92.75	ND	2	0	0.30	0.00	0.15
**P02790|HEMO_HUMAN**	Hemopexin OS=Homo sapiens GN=HPX PE=1 SV=2	51676	77.47	ND	1	0	0.27	0.00	0.14
**P06396|GELS_HUMAN**	Gelsolin OS=Homo sapiens GN=GSN PE=1 SV=1	85697	ND	56.56	0	1	0.00	0.26	0.13
**Q2VWP7|PRTG_HUMAN**	Protogenin OS=Homo sapiens GN=PRTG PE=2 SV=1	127076	ND	30.81	0	1	0.00	0.18	0.09

Table S2d: (4% FAU + 100% human plasma)

Accession	Description	Mw	-10lgP S 1	-10lgP S 2	Spectra S 1	Spectra S 2	NpSpCk S 1	NpSpCk S 2	Average NpSpCk
**P02656|APOC3_HUMAN**	Apolipoprotein C-III OS=Homo sapiens GN=APOC3 PE=1 SV=1	10852	182.39	161.92	11	5	19.14	19.87	19.50
**P02679|FIBG_HUMAN**	Fibrinogen gamma chain OS=Homo sapiens GN=FGG PE=1 SV=3	51512	406.5	259.08	32	15	11.73	12.56	12.14
**P02671|FIBA_HUMAN**	Fibrinogen alpha chain OS=Homo sapiens GN=FGA PE=1 SV=2	94973	365.23	242.46	42	25	8.35	11.35	9.85
**P02768|ALBU_HUMAN**	Serum albumin OS=Homo sapiens GN=ALB PE=1 SV=2	69367	371.46	266.1	33	17	8.98	10.57	9.78
**P02675|FIBB_HUMAN**	Fibrinogen beta chain OS=Homo sapiens GN=FGB PE=1 SV=2	55928	281.18	117.53	20	9	6.75	6.94	6.85
**P01024|CO3_HUMAN**	Complement C3 OS=Homo sapiens GN=C3 PE=1 SV=2	187147	493.22	400.83	52	25	5.25	5.76	5.50
**P19823|ITIH2_HUMAN**	Inter-alpha-trypsin inhibitor heavy chain H2 OS=Homo sapiens GN=ITIH2 PE=1 SV=2	106463	327.72	243.28	18	17	3.19	6.89	5.04
**P02647|APOA1_HUMAN**	Apolipoprotein A-I OS=Homo sapiens GN=APOA1 PE=1 SV=1	30778	89.76	138.35	5	4	3.07	5.60	4.34
**P19827|ITIH1_HUMAN**	Inter-alpha-trypsin inhibitor heavy chain H1 OS=Homo sapiens GN=ITIH1 PE=1 SV=3	101389	379.52	250.9	17	11	3.17	4.68	3.92
**P02649|APOE_HUMAN**	Apolipoprotein E OS=Homo sapiens GN=APOE PE=1 SV=1	36154	274.08	ND	11	0	5.75	0.00	2.87
**P04004|VTNC_HUMAN**	Vitronectin OS=Homo sapiens GN=VTN PE=1 SV=1	54306	266.02	198.53	8	3	2.78	2.38	2.58
**P04114|APOB_HUMAN**	Apolipoprotein B-100 OS=Homo sapiens GN=APOB PE=1 SV=2	515611	408.17	266.27	37	24	1.36	2.01	1.68
**P00747|PLMN_HUMAN**	Plasminogen OS=Homo sapiens GN=PLG PE=1 SV=2	90569	ND	129.45	0	7	0.00	3.33	1.67
**P02749|APOH_HUMAN**	Beta-2-glycoprotein 1 OS=Homo sapiens GN=APOH PE=1 SV=3	38298	94.02	25.49	4	1	1.97	1.13	1.55
**P13671|CO6_HUMAN**	Complement component C6 OS=Homo sapiens GN=C6 PE=1 SV=3	104786	234.37	123.72	9	3	1.62	1.23	1.43
**P01042|KNG1_HUMAN**	Kininogen-1 OS=Homo sapiens GN=KNG1 PE=1 SV=2	71957	217.23	26.78	8	1	2.10	0.60	1.35
**P01857|IGHG1_HUMAN**	Ig gamma-1 chain C region OS=Homo sapiens GN=IGHG1 PE=1 SV=1	36106	107.38	ND	5	0	2.61	0.00	1.31
**P00748|FA12_HUMAN**	Coagulation factor XII OS=Homo sapiens GN=F12 PE=1 SV=3	67792	111.36	54.32	2	3	0.56	1.91	1.23
**P07360|CO8G_HUMAN**	Complement component C8 gamma chain OS=Homo sapiens GN=C8G PE=1 SV=3	22277	54.11	ND	2	0	1.70	0.00	0.85
**P02746|C1QB_HUMAN**	Complement C1q subcomponent subunit B OS=Homo sapiens GN=C1QB PE=1 SV=3	26722	99.52	ND	2	0	1.41	0.00	0.71
**P07358|CO8B_HUMAN**	Complement component C8 beta chain OS=Homo sapiens GN=C8B PE=1 SV=3	67047	157.95	ND	5	0	1.41	0.00	0.70
**P00751|CFAB_HUMAN**	Complement factor B OS=Homo sapiens GN=CFB PE=1 SV=2	85533	139.43	ND	6	0	1.32	0.00	0.66

**P35542|SAA4_HUMAN**	Serum amyloid A-4 protein OS=Homo sapiens GN=SAA4 PE=1 SV=2	14747	78.53	ND	1	0	1.28	0.00	0.64
**P09871|C1S_HUMAN**	Complement C1s subcomponent OS=Homo sapiens GN=C1S PE=1 SV=1	76685	131.93	ND	4	0	0.98	0.00	0.49
**P02765|FETUA_HUMAN**	Alpha-2-HS-glycoprotein OS=Homo sapiens GN=AHSG PE=1 SV=1	39325	159.06	ND	2	0	0.96	0.00	0.48
**P04003|C4BPA_HUMAN**	C4b-binding protein alpha chain OS=Homo sapiens GN=C4BPA PE=1 SV=2	67033	53.15	63.87	1	1	0.28	0.64	0.46
**P03952|KLKB1_HUMAN**	Plasma kallikrein OS=Homo sapiens GN=KLKB1 PE=1 SV=1	71370	184.59	ND	3	0	0.79	0.00	0.40
**Q6NX45|ZN774_HUMAN**	Zinc finger protein 774 OS=Homo sapiens GN=ZNF774 PE=2 SV=2	55068	ND	25.25	0	1	0.00	0.78	0.39
**P08603|CFAH_HUMAN**	Complement factor H OS=Homo sapiens GN=CFH PE=1 SV=4	139096	163.05	ND	5	0	0.68	0.00	0.34
**Q04756|HGFA_HUMAN**	Hepatocyte growth factor activator OS=Homo sapiens GN=HGFAC PE=1 SV=1	70682	ND	90.99	0	1	0.00	0.61	0.31
**P06396|GELS_HUMAN**	Gelsolin OS=Homo sapiens GN=GSN PE=1 SV=1	85697	ND	41.66	0	1	0.00	0.50	0.25
**P00738|HPT_HUMAN**	Haptoglobin OS=Homo sapiens GN=HP PE=1 SV=1	45205	93.02	ND	1	0	0.42	0.00	0.21
**Q8WZ75|ROBO4_HUMAN**	Roundabout homolog 4 OS=Homo sapiens GN=ROBO4 PE=1 SV=1	107457	ND	26.93	0	1	0.00	0.40	0.20
**P00450|CERU_HUMAN**	Ceruloplasmin OS=Homo sapiens GN=CP PE=1 SV=1	122205	68.28	ND	2	0	0.31	0.00	0.15
**B1AJZ9|FHAD1_HUMAN**	Forkhead-associated domain-containing protein 1 OS=Homo sapiens GN=FHAD1 PE=2 SV=2	161903	ND	28.36	0	1	0.00	0.27	0.13
**P02751|FINC_HUMAN**	Fibronectin OS=Homo sapiens GN=FN1 PE=1 SV=4	262622	47.64	ND	1	0	0.07	0.00	0.04

Table S2e: (8% FAU + 100% human plasma)

Accession	Description	Mw	-10lgP		Spectra		NpSpCk		Average NpSpCk
			S 1	S 2	S 1	S 2	S 1	S 2	
**P02656|APOC3_HUMAN**	Apolipoprotein C-III OS=Homo sapiens GN=APOC3 PE=1 SV=1	10852	251.99	222.92	11	7	23.25	28.70	25.97
**P02671|FIBA_HUMAN**	Fibrinogen alpha chain OS=Homo sapiens GN=FGA PE=1 SV=2	94973	386.26	325.21	41	27	9.90	12.65	11.27
**P02768|ALBU_HUMAN**	Serum albumin OS=Homo sapiens GN=ALB PE=1 SV=2	69367	388.33	327.53	25	15	8.27	9.62	8.94
**P02675|FIBB_HUMAN**	Fibrinogen beta chain OS=Homo sapiens GN=FGB PE=1 SV=2	55928	372.25	198.41	23	9	9.43	7.16	8.30
**P02679|FIBG_HUMAN**	Fibrinogen gamma chain OS=Homo sapiens GN=FGG PE=1 SV=3	51512	316.99	231.61	16	7	7.12	6.05	6.58

**P19827|ITIH1_HUMAN**	Inter-alpha-trypsin inhibitor heavy chain H1 OS=Homo sapiens GN=ITIH1 PE=1 SV=3	101389	333.77	273.09	19	15	4.30	6.58	5.44
**P19823|ITIH2_HUMAN**	Inter-alpha-trypsin inhibitor heavy chain H2 OS=Homo sapiens GN=ITIH2 PE=1 SV=2	106463	343	278.81	18	13	3.88	5.43	4.66
**P01024|CO3_HUMAN**	Complement C3 OS=Homo sapiens GN=C3 PE=1 SV=2	187147	382.82	354.57	27	13	3.31	3.09	3.20
**P02647|APOA1_HUMAN**	Apolipoprotein A-I OS=Homo sapiens GN=APOA1 PE=1 SV=1	30778	193.74	109.18	4	2	2.98	2.89	2.94
**P04004|VTNC_HUMAN**	Vitronectin OS=Homo sapiens GN=VTN PE=1 SV=1	54306	200.26	208.7	5	3	2.11	2.46	2.28
**P00751|CFAB_HUMAN**	Complement factor B OS=Homo sapiens GN=CFB PE=1 SV=2	85533	74.75	41.75	7	3	1.88	1.56	1.72
**P35542|SAA4_HUMAN**	Serum amyloid A-4 protein OS=Homo sapiens GN=SAA4 PE=1 SV=2	14747	120.98	ND	2	0	3.11	0.00	1.56
**P02748|CO9_HUMAN**	Complement component C9 OS=Homo sapiens GN=C9 PE=1 SV=2	63173	133.39	74.38	4	2	1.45	1.41	1.43
**P00748|FA12_HUMAN**	Coagulation factor XII OS=Homo sapiens GN=F12 PE=1 SV=3	67792	116.88	73.86	4	2	1.35	1.31	1.33
**P01857|IGHG1_HUMAN**	Ig gamma-1 chain C region OS=Homo sapiens GN=IGHG1 PE=1 SV=1	36106	100.75	ND	4	0	2.54	0.00	1.27
**P02749|APOH_HUMAN**	Beta-2-glycoprotein 1 OS=Homo sapiens GN=APOH PE=1 SV=3	38298	59.18	34.52	2	1	1.20	1.16	1.18
**P02765|FETUA_HUMAN**	Alpha-2-HS-glycoprotein OS=Homo sapiens GN=AHSG PE=1 SV=1	39325	194.74	ND	4	0	2.33	0.00	1.17
**P02760|AMBP_HUMAN**	Protein AMBP OS=Homo sapiens GN=AMBP PE=1 SV=1	39000	82.5	78.41	2	1	1.18	1.14	1.16
**P07358|CO8B_HUMAN**	Complement component C8 beta chain OS=Homo sapiens GN=C8B PE=1 SV=3	67047	183.29	63.79	4	1	1.37	0.66	1.02
**P0C0L4|CO4A_HUMAN**	Complement C4-A OS=Homo sapiens GN=C4A PE=1 SV=1	192770	266.75	148.38	7	4	0.83	0.92	0.88
**P0C0L5|CO4B_HUMAN**	Complement C4-B OS=Homo sapiens GN=C4B PE=1 SV=1	192792	266.75	148.38	7	4	0.83	0.92	0.88
**P09871|C1S_HUMAN**	Complement C1s subcomponent OS=Homo sapiens GN=C1S PE=1 SV=1	76685	ND	123.77	0	3	0.00	1.74	0.87
**P01861|IGHG4_HUMAN**	Ig gamma-4 chain C region OS=Homo sapiens GN=IGHG4 PE=1 SV=1	35941	ND	36.89	0	1	0.00	1.24	0.62
**P08603|CFAH_HUMAN**	Complement factor H OS=Homo sapiens GN=CFH PE=1 SV=4	139096	207.92	ND	6	0	0.99	0.00	0.49
**P51957|NEK4_HUMAN**	Serine/threonine-protein kinase Nek4 OS=Homo sapiens GN=NEK4 PE=1 SV=2	94597	28.94	ND	4	0	0.97	0.00	0.48
**P01042|KNG1_HUMAN**	Kininogen-1 OS=Homo sapiens GN=KNG1 PE=1 SV=2	71957	141.21	ND	3	0	0.96	0.00	0.48
**P02790|HEMO_HUMAN**	Hemopexin OS=Homo sapiens GN=HPX PE=1 SV=2	51676	44.97	ND	2	0	0.89	0.00	0.44
**P15169|CBPN_HUMAN**	Carboxypeptidase N catalytic chain OS=Homo sapiens GN=CPN1 PE=1 SV=1	52286	ND	38.74	0	1	0.00	0.85	0.43
**P07357|CO8A_HUMAN**	Complement component C8 alpha chain OS=Homo sapiens GN=C8A PE=1 SV=2	65163	116.85	ND	2	0	0.70	0.00	0.35

**O94819|KBTBB_HUMAN**	Kelch repeat and BTB domain-containing protein 11 OS=Homo sapiens GN=KBTBD11 PE=1 SV=1	65720	ND	36.41	0	1	0.00	0.68	0.34
**P02649|APOE_HUMAN**	Apolipoprotein E OS=Homo sapiens GN=APOE PE=1 SV=1	36154	66.56	ND	1	0	0.63	0.00	0.32
**P00736|C1R_HUMAN**	Complement C1r subcomponent OS=Homo sapiens GN=C1R PE=1 SV=2	80119	ND	36.64	0	1	0.00	0.56	0.28
**P00738|HPT_HUMAN**	Haptoglobin OS=Homo sapiens GN=HP PE=1 SV=1	45205	53.79	ND	1	0	0.51	0.00	0.25
**P06727|APOA4_HUMAN**	Apolipoprotein A-IV OS=Homo sapiens GN=APOA4 PE=1 SV=3	45399	88.46	ND	1	0	0.51	0.00	0.25
**P04114|APOB_HUMAN**	Apolipoprotein B-100 OS=Homo sapiens GN=APOB PE=1 SV=2	515611	239.94	99.75	6	2	0.27	0.17	0.22
**Q8IXZ2|ZC3H3_HUMAN**	Zinc finger CCCH domain-containing protein 3 OS=Homo sapiens GN=ZC3H3 PE=1 SV=3	101941	ND	26.31	0	1	0.00	0.44	0.22
**P53420|CO4A4_HUMAN**	Collagen alpha-4(IV) chain OS=Homo sapiens GN=COL4A4 PE=1 SV=3	164037	29.55	ND	3	0	0.42	0.00	0.21
**Q9HC56|PCDH9_HUMAN**	Protocadherin-9 OS=Homo sapiens GN=PCDH9 PE=1 SV=2	136064	ND	26.91	0	1	0.00	0.33	0.16
**P03952|KLKB1_HUMAN**	Plasma kallikrein OS=Homo sapiens GN=KLKB1 PE=1 SV=1	71370	61.36	ND	1	0	0.32	0.00	0.16
**Q7Z5J8|ANKAR_HUMAN**	Ankyrin and armadillo repeat-containing protein OS=Homo sapiens GN=ANKAR PE=2 SV=3	162025	ND	29.35	0	1	0.00	0.27	0.14
**P02751|FINC_HUMAN**	Fibronectin OS=Homo sapiens GN=FN1 PE=1 SV=4	262622	25.48	ND	1	0	0.09	0.00	0.04
**Q6UB99|ANR11_HUMAN**	Ankyrin repeat domain-containing protein 11 OS=Homo sapiens GN=ANKRD11 PE=1 SV=3	297911	25.25	ND	1	0	0.08	0.00	0.04
**Q96JG9|ZN469_HUMAN**	Zinc finger protein 469 OS=Homo sapiens GN=ZNF469 PE=1 SV=3	410207	24.72	ND	1	0	0.06	0.00	0.03

Table S2f: (12% FAU + 100% human plasma)

Accession	Description	Mw	-10lgP S 1	-10lgP S 2	Spectra S 1	Spectra S 2	NpSpCk S 1	NpSpCk S 2	Average NpSpCk
**P02656|APOC3_HUMAN**	Apolipoprotein C-III OS=Homo sapiens GN=APOC3 PE=1 SV=1	10852	259.65	263.71	12	6	22.68	23.56	23.12
**P02679|FIBG_HUMAN**	Fibrinogen gamma chain OS=Homo sapiens GN=FGG PE=1 SV=3	51512	384.9	282.85	27	11	10.75	9.10	9.92
**P02671|FIBA_HUMAN**	Fibrinogen alpha chain OS=Homo sapiens GN=FGA PE=1 SV=2	94973	367.67	281.85	43	23	9.29	10.32	9.80
**P02768|ALBU_HUMAN**	Serum albumin OS=Homo sapiens GN=ALB PE=1 SV=2	69367	372.84	333.91	34	15	10.05	9.21	9.63
**P02675|FIBB_HUMAN**	Fibrinogen beta chain OS=Homo sapiens GN=FGB PE=1 SV=2	55928	340.52	209.02	22	11	8.07	8.38	8.22
**P19827|ITIH1_HUMAN**	Inter-alpha-trypsin inhibitor heavy chain H1 OS=Homo sapiens GN=ITIH1 PE=1 SV=3	101389	378.57	407.28	28	21	5.66	8.82	7.24
**P19823|ITIH2_HUMAN**	Inter-alpha-trypsin inhibitor heavy chain H2 OS=Homo sapiens GN=ITIH2 PE=1 SV=2	106463	365.4	346.93	21	22	4.05	8.80	6.42
**P04004|VTNC_HUMAN**	Vitronectin OS=Homo sapiens GN=VTN PE=1 SV=1	54306	251.16	262.66	10	5	3.78	3.92	3.85
**P02647|APOA1_HUMAN**	Apolipoprotein A-I OS=Homo sapiens GN=APOA1 PE=1 SV=1	30778	166.81	161.68	5	2	3.33	2.77	3.05
**P00747|PLMN_HUMAN**	Plasminogen OS=Homo sapiens GN=PLG PE=1 SV=2	90569	233.27	171.78	8	8	1.81	3.76	2.79
**P01024|CO3_HUMAN**	Complement C3 OS=Homo sapiens GN=C3 PE=1 SV=2	187147	426.87	386.41	20	12	2.19	2.73	2.46
**P02649|APOE_HUMAN**	Apolipoprotein E OS=Homo sapiens GN=APOE PE=1 SV=1	36154	170.95	ND	4	0	2.27	0.00	1.13
**P02760|AMBP_HUMAN**	Protein AMBP OS=Homo sapiens GN=AMBP PE=1 SV=1	39000	ND	58.57	0	2	0.00	2.18	1.09
**P0C0L4|CO4A_HUMAN**	Complement C4-A OS=Homo sapiens GN=C4A PE=1 SV=1	192770	203.76	143.77	8	5	0.85	1.11	0.98
**P0C0L5|CO4B_HUMAN**	Complement C4-B OS=Homo sapiens GN=C4B PE=1 SV=1	192792	203.76	143.77	8	5	0.85	1.10	0.98
**P01042|KNG1_HUMAN**	Kininogen-1 OS=Homo sapiens GN=KNG1 PE=1 SV=2	71957	135.73	78.39	4	1	1.14	0.59	0.87
**P02765|FETUA_HUMAN**	Alpha-2-HS-glycoprotein OS=Homo sapiens GN=AHSG PE=1 SV=1	39325	183.38	ND	3	0	1.56	0.00	0.78
**P00450|CERU_HUMAN**	Ceruloplasmin OS=Homo sapiens GN=CP PE=1 SV=1	122205	134.61	71.87	5	2	0.84	0.70	0.77
**P04114|APOB_HUMAN**	Apolipoprotein B-100 OS=Homo sapiens GN=APOB PE=1 SV=2	515611	232.58	238.32	12	12	0.48	0.99	0.73
**P35542|SAA4_HUMAN**	Serum amyloid A-4 protein OS=Homo sapiens GN=SAA4 PE=1 SV=2	14747	124.34	ND	1	0	1.39	0.00	0.70
**P07358|CO8B_HUMAN**	Complement component C8 beta chain OS=Homo sapiens GN=C8B PE=1 SV=3	67047	160.9	ND	4	0	1.22	0.00	0.61
**P00751|CFAB_HUMAN**	Complement factor B OS=Homo sapiens GN=CFB PE=1 SV=2	85533	98.7	ND	5	0	1.20	0.00	0.60
**P04196|HRG_HUMAN**	Histidine-rich glycoprotein OS=Homo sapiens GN=HRG PE=1 SV=1	59578	45.77	ND	3	0	1.03	0.00	0.52

**P07360|CO8G_HUMAN**	Complement component C8 gamma chain OS=Homo sapiens GN=C8G PE=1 SV=3	22277	94.8	ND	1	0	0.92	0.00	0.46
**P00738|HPT_HUMAN**	Haptoglobin OS=Homo sapiens GN=HP PE=1 SV=1	45205	36.07	ND	2	0	0.91	0.00	0.45
**P13671|CO6_HUMAN**	Complement component C6 OS=Homo sapiens GN=C6 PE=1 SV=3	104786	ND	56.49	0	2	0.00	0.81	0.41
**Q9H6K5|YS027_HUMAN**	Putative uncharacterized protein FLJ22184 OS=Homo sapiens PE=1 SV=1	60039	35.56	ND	2	0	0.68	0.00	0.34
**P04003|C4BPA_HUMAN**	C4b-binding protein alpha chain OS=Homo sapiens GN=C4BPA PE=1 SV=2	67033	ND	31.62	0	1	0.00	0.64	0.32
**P00748|FA12_HUMAN**	Coagulation factor XII OS=Homo sapiens GN=F12 PE=1 SV=3	67792	90.73	ND	2	0	0.61	0.00	0.30
**P08603|CFAH_HUMAN**	Complement factor H OS=Homo sapiens GN=CFH PE=1 SV=4	139096	111.35	ND	4	0	0.59	0.00	0.29
**P02749|APOH_HUMAN**	Beta-2-glycoprotein 1 OS=Homo sapiens GN=APOH PE=1 SV=3	38298	72.34	ND	1	0	0.54	0.00	0.27
**P06396|GELS_HUMAN**	Gelsolin OS=Homo sapiens GN=GSN PE=1 SV=1	85697	ND	36.85	0	1	0.00	0.50	0.25
**Q14410|GLPK2_HUMAN**	Glycerol kinase 2 OS=Homo sapiens GN=GK2 PE=2 SV=2	60594	34.37	ND	1	0	0.34	0.00	0.17
**P00734|THRB_HUMAN**	Prothrombin OS=Homo sapiens GN=F2 PE=1 SV=2	70037	64.19	ND	1	0	0.29	0.00	0.15
**P00736|C1R_HUMAN**	Complement C1r subcomponent OS=Homo sapiens GN=C1R PE=1 SV=2	80119	79.24	ND	1	0	0.26	0.00	0.13
**P49815|TSC2_HUMAN**	Tuberin OS=Homo sapiens GN=TSC2 PE=1 SV=2	200607	27.34	ND	2	0	0.20	0.00	0.10
**Q96L96|ALPK3_HUMAN**	Alpha-protein kinase 3 OS=Homo sapiens GN=ALPK3 PE=2 SV=2	201270	37.91	ND	1	0	0.10	0.00	0.05
**P02751|FINC_HUMAN**	Fibronectin OS=Homo sapiens GN=FN1 PE=1 SV=4	262622	42.55	ND	1	0	0.08	0.00	0.04

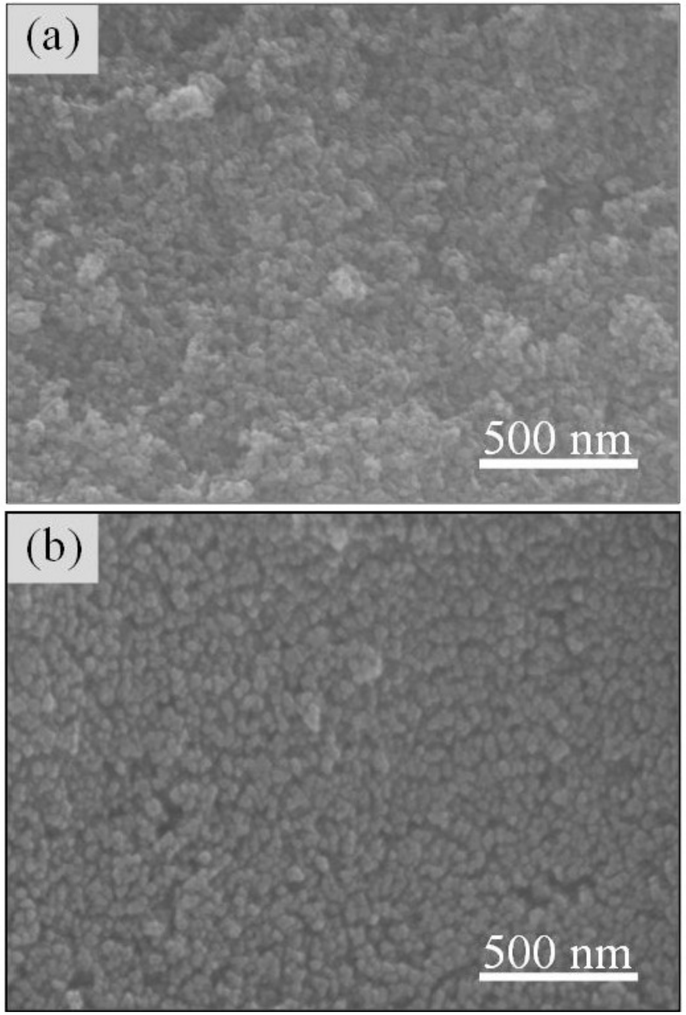

Figure Sl. SEM images of(a) EMT, and (b) FAU zeolite NPs.

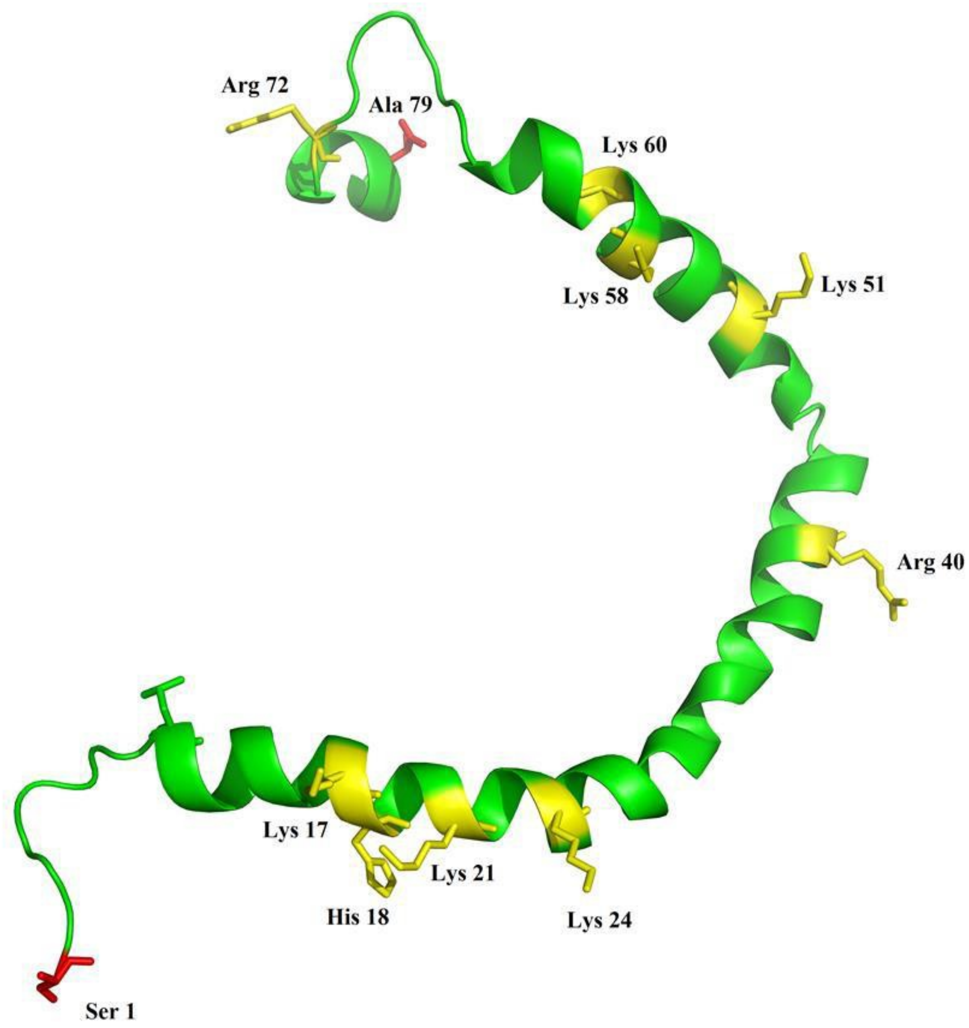

Figure S2. Ribbon diagram of apolipoprotein C-III (APOC-III, PDB entry: 2JQ3) adsorbed on the EMT NPs. All positively charged amino acid residues, distributed through the structure of APOC-III are colored in yellow. N-terminal *Ser-1* and C-terminal *Ala-79* are marked in red. Hydrogen is removed from the structure for better visualization.
